# Quaternary Ammonium Salts of Cationic Lipopeptides with Lysine Residues — Synthesis, Antimicrobial, Hemolytic and Cytotoxic Activities

**DOI:** 10.1007/s12602-023-10161-8

**Published:** 2023-09-29

**Authors:** Karol Sikora, Jakub Jędrzejczak, Marta Bauer, Damian Neubauer, Maciej Jaśkiewicz, Magdalena Szaryńska

**Affiliations:** 1 Department of Inorganic Chemistry, Faculty of Pharmacy, Medical University of Gdańsk, Al. Gen. J. Hallera 107, 80-416 Gdańsk, Poland; 2 International Research Agenda 3P- Medicine Laboratory, Medical University of Gdańsk, Dębinki 7, Building no. 5, 80-211 Gdańsk, Poland; 3 Department of Histology, Faculty of Medicine, Medical University of Gdańsk, Dębinki 1, 80-211 Gdańsk, Poland

**Keywords:** Ultrashort cationic lipopeptides, Quaternary ammonium salts, Synthesis, ESKAPE, Antimicrobial, Hemolytic, Cytotoxicity

## Abstract

Ultrashort cationic lipopeptides (USCLs) and quaternary ammonium salts constitute two groups of cationic surfactants with high antimicrobial activity. This study aimed to investigate the influence of quaternization of the amino group of the lysine side chain in USCLs on their antimicrobial, hemolytic and cytotoxic activities. To do this, two series of lipopeptides were synthesized, USLCs and their quaternized analogues containing trimethylated lysine residues — qUSCLs (quaternized ultrashort cationic lipopeptides). Quaternization was performed on a resin during a standard solid-phase peptide synthesis with CH_3_I as the methylating agent. According to our knowledge, this is the first study presenting on-resin peptide quaternization. The lipopeptides were tested for their antibacterial and antifungal activities against the ESKAPE group (*Enterococcus faecium, Staphylococcus aureus, Klebsiella pneumoniae, Acinetobacter baumannii, Pseudomonas aeruginosa* and *Klebsiella aerogenes*) bacteria and *Candida glabrata* yeast-like fungus. Most of the compounds proved to be active antimicrobial agents with enhanced activity against Gram-positive strains and fungi and a lower against Gram-negative species. In addition, the antimicrobial activity of lipopeptides was increasing with an increase in hydrophobicity but qUSCLs exhibited usually a poorer antimicrobial activity than their parent molecules. Furthermore, the toxicity against red blood cells and human keratinocytes was assessed. It’s worth emphasizing that qUSCLs were less toxic than the parent molecules of comparative hydrophobicity. The results of the study proved that qUSCLs can offer a higher selectivity to pathogens over human cells than that of USCLs. Last but not least, quaternization of the peptides could increase their solubility and therefore their bioavailability and utility.

**Supplementary Information:**

The online version contains supplementary material available at 10.1007/s12602-023-10161-8.

## Introduction

Dramatically increasing antimicrobial resistance force to develop new antibiotics. In 2020, WHO stated that antibiotic resistance is one of the biggest threats to global health. Increasing antibiotic resistance leads to longer hospitalization, higher medical costs and increased mortality. Moreover, WHO strongly support research of new antimicrobial agents and therapies [1]. In response to this global issue, the WHO, published a priority pathogen list, named ESKAPE strains, for which new antibiotics for the treatment and prevention of infections are highly demanded. The group of ESKAPE pathogens comprises clinically important Gram-negative and Gram-positive bacteria with multidrug resistance and high virulence. ESKAPE bacteria are the following: *Acinetobacter baumannii*, *Enterococcus faecium*, *Klebsiella aerogenes* (formerly *Enterobacter aerogenes*), *Klebsiella pneumonia*, *Pseudomonas aeruginosa* and *Staphylococcus aureus* [2]. Ultrashort cationic lipopeptides (USCLs) represent an interesting class of surfactants that are promising candidates for new antimicrobial drugs owing to their extraordinary antibacterial and antifungal activities. USCLs contain up to seven amino acid residues and at least one lipophilic group. Typically, these are fatty acids attached to the *N*-terminal *N*^*α*^-amino group. Usually, USCLs contain lysine, arginine or histidine and as a consequence of their basicity at pHs below pK_a_ of amino groups, they are in ionised/protonated form with a positive net charge. USCLs exhibit a broad spectrum of antibacterial and antifungal activity with low propensity to induce resistance, but a serious drawback of this class is a relatively high toxicity to human cells and low selectivity limiting their applicability. However, there are numerous studies regarding improvement of selectivity of USCLs accompanied with decreased toxicity. For example, superiority in terms of lowered toxicity towards human keratinocytes, of cyclic USCL, C_16_-CRRKKC-NH_2_, with disulfide bridge over its linear analogue, C_16_-CRRKKC-NH_2_, was reported by our group. IC_50_ values for C_16_-RRKK-NH_2_ and its cyclic analogue, C_16_-CRRKKC-NH_2_ were 8.1 ± 1.1 and 21.3 ± 8.2 respectively [3]. It was found that the cationic net charge and the length of the lipid chain were crucial for the biological activity of USCLs [4]. Moreover, numerous studies revealed that in the case of each USCLs series, there was an optimum within hydrophobicity of a compound of the highest selectivity between pathogens and normal human cells. The balance between hydrophobic (fatty acid) and hydrophilic (peptide chain) parts is crucial for selectivity [5–7]. For ornithine and tryptophan based USCLs with C_6_ to C_16_ fatty acids, highest antimicrobial activity exhibited USCLs with C_12_ and C_14_ fatty acids. For example, MIC for C_12_-Orn-Orn-Trp-Trp-NH_2_ was in range of 0.95–1.95 μg/mL against *S. aureus*, while significant cytotoxicity at concentrations greater than 62.5 μg/mL was observed. Those results suggested high selectivity of this lipopetide [5]. Therefore, in the case of a new group of USCLs, it is necessary to include lipid substituents of different chain lengths or different numbers of cationic residues. Appropriate diversity of a set of compounds can provide sufficient data to determine the structure/activity relationship. Similarly, the type of the basic amino acid residue can affect USCLs characteristics as well as that of regular antimicrobial peptides [3, 8, 9]. Studies on introduction of *N*^*ε*^-trimethylated lysine which is quaternary ammonium salt revealed that this modification could lead to peptides with improved proteolytic stability and selectivity between bacteria and human cells [10]. Quaternary ammonium salts (QASs) possess in their structure a nitrogen atom with a positive charge and four organic substituents attached to this atom, where one of them is hydrophobic. Generally, QASs have been recognized as surfactants and antimicrobial agents owing to their high activity against bacteria and fungi. One of the examples of commonly used antimicrobial agent is benzalkonium chloride (BAC). It possess broad-spectrum of antimicrobial properties and due to that it is commonly used in industry, household and hospitals as active ingredient of disinfectant agents [11]. However, QASs applications are not only limited to disinfectants, they found multiple applications in almost any area of modern society. For instance in medicine they are used as muscle relaxants used in anesthesia (D-tubocurarine and dimethyltubocurarine) and treatment of pulmonary disorders (ipratropium bromide). In pharmaceutical formulations as preservatives (BAC in ophthalmic and nasal solutions) or components of dental materials to prevent dental caries [12–15]. Moreover, QASs are used in agriculture as pesticides and herbicides (paraquat, diquat), households (fabric softeners), industry (catalysis, corrosion inhibitors, ionic liquids, electrolytes) and laboratories (asymmetric synthesis, chiral resolution, etc.) [16–20]. A specific subclass of QASs arises from modified amino acids and peptides. Lysine is one of the dominating amino acids modified by quaternization, the main reason is the fact that it possesses two amine groups, attached to the α and ε carbon atoms, thus allowing multiple modification combinations. Other amino acids including arginine, histidine or phenylalanine were also used; however, *N*^*α*^ atom is quaternized in those amino acids. Quaternization of side chain histidine was also reported; however, lysine seems to be a better platform for development of new compounds due to fact that side chain amine group is primary amine and is easily accessed by other molecules due to smaller steric hindrance, thus it can be easily modified. Furthermore, *N*^*ε*^-trimethyllysine can be found in some proteins as one of posttranslational modifications [21–26]. One of examples of amino acids quaternization was reported by Colomer et al. [21, 22]. The authors presented synthesis, physicochemical and antimicrobial properties of lysine and arginine-based cationic surfactants. Synthesized compounds represented monomeric and dimeric (gemini) surfactants with hydrophobic residue (lauric acid) attached to the amine group of the lysine side chain, moreover, part of these compounds were QAS with *N*^*α*^ atom substituted with at least three methylene groups. Among all compounds, the highest antimicrobial activity was observed for monomeric and dimeric surfactants containing the quaternized amine group. One of the well-studied USCLs with documented exceptional antimicrobial activity is C_16_-KKKK-NH_2_ [3, 27–29]. Nevertheless, its main shortcoming is a distinct toxicity that deteriorates its selectivity. Bearing in mind that QAS possess high selectivity, it suggests that USCLs toxicity and non-selectivity can be overcome through lysine quaternization. This hypothesis was made based on Fernandez-Reyes et al. study on antimicrobial peptide (cecropin A−melittin hybrid) [10]. The authors suggest that lysine *N*^ε^-Trimethylation can be a tool for improving the selectivity of peptides. Therefore, quaternary ammonium USCLs (qUSCLs) can have elevated selectivity as compared to that of parent molecules.

**Fig. 1:**
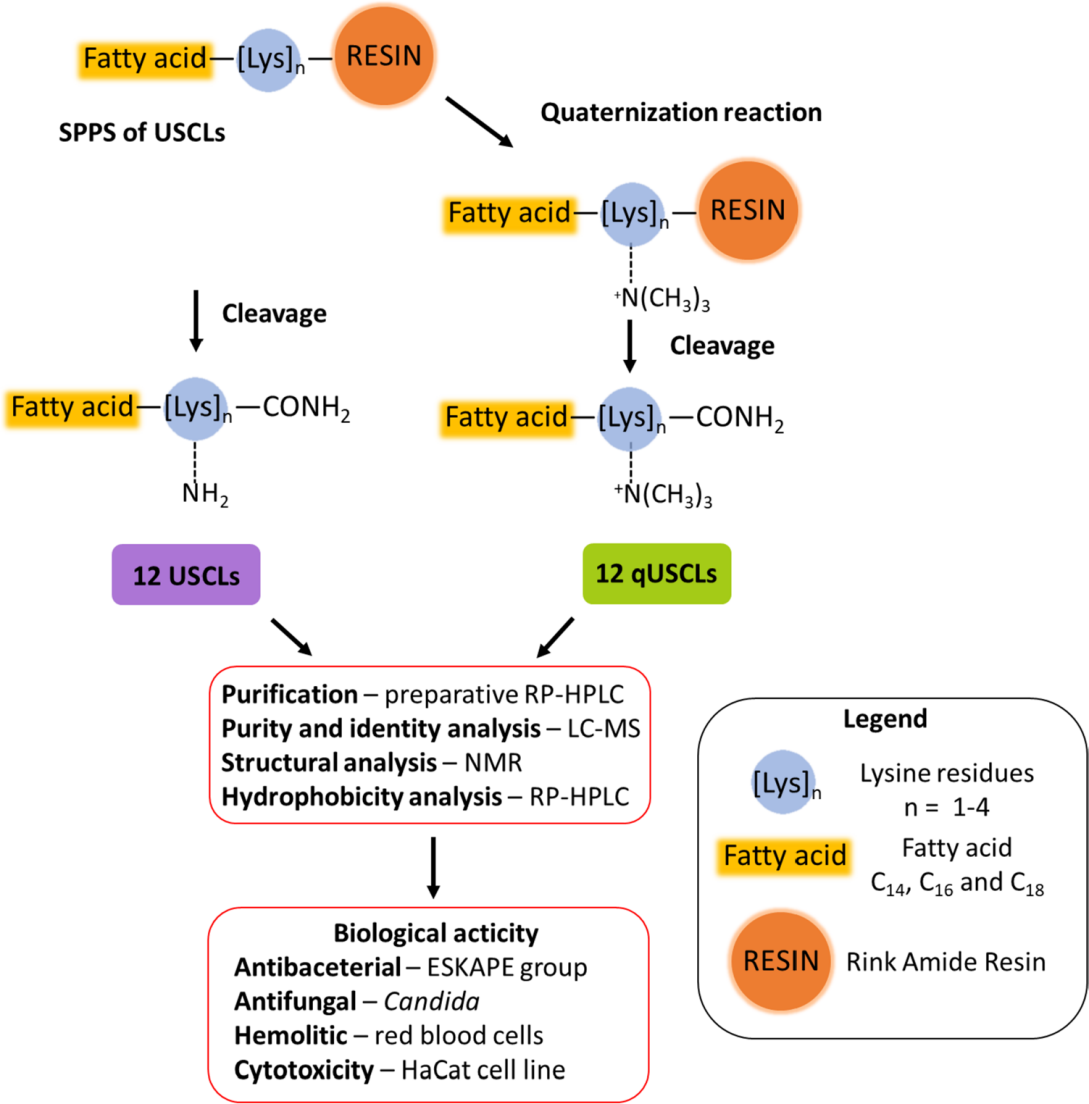
Research plan of synthesis and biological activity evaluation of USCLs and qUSCLs

This study aimed to verify the effect of lysine *N*^*ε*^-trimethylation of USCLs differing in the number of lysine residues (between 1 and 4) and the length of the *N*-terminal fatty acid chain on biological characteristics. One group of investigated compounds were USCLs containing lysine residues (1, 2, 3, or 4) and one fatty acid residue (myristic — C_14_, palmitic — C_16_, or stearic — C_18_). The other group were USCLs analogs with *N*^*ε*^-trimethylated amino groups in each side-chain of lysine residues (qUSCLs). As a result, 12 USCLs and 12 qUSCLs were synthetized. Moreover, we have elaborated an on-resin method of the synthesis of qUSCLs using SPPS (solid-phase peptide synthesis) with CH_3_I as methylating agent. Importantly, quaternization was performed after coupling of all amino acids and fatty acid residue. After the synthesis, all USCLs and qUSCLs were tested in vitro for their antibacterial (against ESKAPE pathogens — *A. baumannii ATCC BAA 1605, E. faecium ATCC 700221, K. aerogenes ATCC 13048 (formerly E. aerogenes), K. pneumoniae ATCC 700603, P. aeruginosa ATCC 9027 and S. aureus ATCC 33591*), antifungal (*Candida glabrata ATCC 15126*), hemolytic and cytotoxic activities (HaCaT cell line) (Fig. 1). Furthermore, selectivity indexes were calculated based on determined minimum inhibitory concentrations (MICs) and HC_50_/IC_50_ (lipopeptide concentration causing 50% hemolysis/of cell growth inhibition). Numerous studies indicate that biological properties of lipopeptides depend on hydrophobicity [6, 7, 30]. Therefore, to evaluate peptides hydrophobicity, RP-HPLC analyses were performed. This is the first study presenting on-resin peptide quaternization and the effect of lysine quaternization in USCLs that differ in charge (between +1 and +4) on its biological activity.

## Materials and Methods

### Synthesis of Lipopeptides

All lipopeptides were synthesized manually by solid-phase peptide synthesis (SPPS) method using the Fmoc/t-bu approach [31, 32] on polystyrene resin modified by a Rink amide linker (4-(2′, 4′-Dimethoxyphenyl-Fmoc-aminomethyl)-phenoxymethyl resin, particle size 100–200 mesh, loading 0.67 mmol/g, crosslinking degree 1% divinylbenzene; Sunresin, China). Deprotection of the Fmoc groups was carried out in a 20% (v/v) piperidine (Merck, Darmstadt, Germany) solution in DMF (*N,N*-dimethylformamide; Honeywell, Seelze, Germany) with constant shaking for 15 min at room temperature. Attachment of protected amino acids was conducted in a DMF/DCM solution (1:1, v/v, DCM – dichloromethane; Chempur, Poland) with coupling agents using a threefold molar excess of DIC (*N,N*′-diisopropylcarbodiimide; Peptideweb, Poland) and OxymaPure (ethyl 2-cyano-2-(hydroxyimino)acetate, Iris Biotech GmbH, Germany) with constant shaking for 1.5 h at room temperature. Therefore, acylation was conducted with a mixture of DIC:OxymaPure:Fmoc-AA-OH (molar ratio 1:1:1) at concentration of 0.1 M. *N*α-Fmoc-protected amino acids were obtained from Carbolution Chemicals GmbH (Germany). All reactions including acylation and deportation were performed in glass reaction vessels and shaken using a Kamush peptide shaker (Kamush, Poland). To remove by-products and excess of reagents from resin, after acylation or deportation steps, the resin was rinsed with DMF (3x) and DCM (3x). Briefly, reaction mixture was removed by suction through reaction vessel frit, next proper solvent was added (DMF or DCM) and mixture was shaken for 2 min and the solvent was removed by suction. Chloranil test was used to control acylation and deprotection processes. Briefly, a few mg of resin were placed in a small test tube. Next, 1 drop of 2% acetaldehyde (Sigma-Aldrich Chemie GmbH, Switzerland) in DMF and 1 drop of 2% p-chloranil (Merck KGaA, Germany) in DMF were added and incubated at room temperature for 5 min. If the beads were blue, free amine groups were present and the coupling reaction was not complete. In such case, second coupling was performed. If the beads remained unstained, coupling reaction was completed. After the synthesis, the peptide resins were dried under vacuum. The peptides were cleaved from the resin using the mixture of TFA (trifluoroacetic acid; Apollo Scientific, UK), 1,3-dimethoxybenzene (Sigma-Aldrich, MO, USA), triisopropylsilane (TIS) (Sigma-Aldrich, MO, USA), and deionized water (92.5:2.5:2.5:2.5 v/v). Cleavage from the resin was accomplished for 1.5 h with agitation. Crude peptides were precipitated with cold diethyl ether (Chempur, Poland) and centrifuged (3461 × g, 5 min; EBA 20, Hettich, Andreas Hettich GmbH & Co. KG, Germany). The supernatant was discarded, and the crude peptide was dissolved in deionized water, frozen with liquid nitrogen and lyophilized using freeze dryer (Christ, Alpha 2-4 ld plus). Samples were lyophilized over 24 h and freeze dryer parameters were vacuum at 0.05 mbar and ice condenser temperature −80 °C. Purification of the compounds was carried out by RP-HPLC.

### On Resin Quaternization of Lipopeptides

Before the quaternization reaction, the resin with attached lipopeptides was treated with 10% TFA in DCM over 30 min to remove Boc (*tert*-butoxycarbonyl) protecting groups from the lysine side chains. Next, the resin was washed 3 times with DCM and 3 times with dry methanol (Sigma-Aldrich, Germany). Next, 0.5 g of KHCO_3_, 5 mL of dry methanol and 0.5 mL of CH_3_I (Sigma-Aldrich, Germany) were added to the resin and the mixture was heated at 70 °C for 3 h. The resin was washed 3 times with MeOH. The final products were cleaved and purified analogously to lipopeptides.

### Purification of Lipopeptides and QAS Lipopeptides

Purifications were carried out by preparative reversed-phase high-performance liquid chromatography (RP-HPLC) on a Phenomenex Gemini-NX C18 column (21.20 × 100 mm, 5.0 µm particle size, 110 Å pore size) using ECOM (Czech Republic) preparative HPLC composed of IOTA-S 100 pumps and Flash 10 DAD UV detector. UV detection at 214 nm was used, and the crude peptides were eluted with a linear 10–70% acetonitrile gradient in deionized water over 90 min at room temperature. The mobile phase flow rate was 10.0 mL/min. Acetonitrile (ACN; HPLC-gradient grade; POCH, Poland) and water, both containing 0.1% of TFA, were used as a mobile phase. Fractions were collected and analyzed by LC-MS. Pure fractions (>95%) were collected and lyophilized.

### Analyses of Purity and Identity of USCLs and qUSCLs

The purity and identity of the peptides were confirmed by HPLC-MS analyses. RP-HPLC system was used — Waters Alliance e2695 system with Waters 2998 PDA and Acquity QDA detectors (software — Empower^®^3). All analyses were carried out on a Waters XBridge™ Shield RP-18 column (4.6 × 150 mm, 3.5 µm particle size, 130 Å pore size). Samples (10 µL) were analyzed with a linear 10–90% acetonitrile gradient in deionized water over 15 min at 25.0 ± 0.1 °C. The mobile phase flow rate was 0.5 mL/min. Both eluents contained 0.1% (v/v) of formic acid. Mass analysis and UV detection at 214 nm were used. MS detector was operated in full scan mode and parameters are as follows: probe temperature 600 °C, Source temperature 110 °C, cone voltage 3 V, capillary voltage — 0.8 kV and mass range 50—1250 Da.

### NMR Analysis of 1M and 1Mq

The structure of peptides **1M** and **1Mq** was confirmed by ^1^H NMR, ^13^C NMR, HSQC and COSY analysis, on Bruker AVANCE III (Bruker, Billerica, Massachusetts, USA). Samples of **1M** and **1Mq** were dissolved in CD_3_OD and the concentration was 10 mg/mL. Spectra were recorded at 500 MHz and 125.76 MHz frequencies for ^1^H and ^13^C NMR respectively. COSY was recorded with Resolution: F1 – 128 and F2 2048 and spectral width 8.401 ppm and 4201.68 Hz. HSQC was recorded with Resolution: F1 – 256 and F2 2048 and spectral width: F1 – 165.00 ppm and 20751.48 Hz F2 – 12.01 ppm and 6009.61 Hz. Ninety-degree pulse was established to obtain best signal to noise ratio.

**C**_**14**_**-K-NH**_**2**_ **(1M)** in CD_3_OD. ^1^H NMR (500 MHz): 4.36 (2 H, dd, *J* 5.4 Hz, J 8.8 Hz, Lys C_α_H); 2.93 (2 H, m, Lys C_ε_H_2_); 1.85 (1 H, m, Lys C_β_H_a_); 1.71 (2 H, m, Lys C_δ_H_2_); 1.69 (1 H, m, Lys C_β_H_b_); 1.47 (2 H, m, Lys C_γ_H_2_). Myristic acid: 2.27 (2 H, dt, *J* 1.1 Hz, J 7.3 Hz); 1.64 (2 H, m); 1.34 (20 H, m); 0.92 (2 H, t, *J* 6.8 Hz). ^13^C NMR (125.76 MHz): 174.99 (C=O, Lys); 52.48 (C_α_, Lys); 39.09 (C_ε_, Lys); 31.66 (C_β_, Lys); 26.68 (C_δ_, Lys); 22.40 (C_γ_, Lys). Myristic acid: 175.46 (C=O, Mir); 35.47 (CH_2_, Mir); 31.12 (CH_2_, Mir); 29.39 (CH_2_, Mir); 29.35 (CH_2_, Mir); 29.24 (CH_2_, Mir); 29.06 (CH_2_, Mir); 28.97 (CH_2_, Mir); 25.47 (CH_2_, Mir); 22.32 (CH_2_, Mir); 13.02 (CH_3_, Mir).

**C**_**14**_**-K(Me**_**3**_**)**^**+**^**-NH**_**2**_ **(1Mq)** in CD_3_OD. ^1^H NMR (500 MHz): 4.35 (2 H, dd, *J* 5.4 Hz, J 8.8 Hz, Lys C_α_H); 3.35 (2 H, m, Lys C_ε_H_2_); 3.15 (8 H, s, N^+^(CH_3_)_3_); 1.87 (1 H, m, Lys C_β_H_a_); 1.85 (2 H, m, Lys C_δ_H_2_); 1.75 (1 H, m, Lys C_β_H_b_); 1.45 (2 H, m, Lys C_γ_H_2_). Myristic acid: 2.28 (2 H, dt, *J* 1.7 Hz, J 7.3 Hz); 1.64 (2 H, m); 1.39 (20 H, m); 0.90 (2 H, t, *J* 6.8 Hz). ^13^C NMR (125.76 MHz): 174.97 (C=O, Lys); 52.35 (C_α_, Lys); 66.11 (C_ε_, Lys); 52.26 (N^+^(CH_3_)_3_); 31.14 (C_β_, Lys); 22.20 (C_γ_, Lys); 21.94 (C_δ_, Lys). Myristic acid: 175.31 (C=O, Mir); 35.49 (CH_2_, Mir); 31.66 (CH_2_, Mir); 29.38 (CH_2_, Mir); 29.35 (CH_2_, Mir); 29.24 (CH_2_, Mir); 29.08 (CH_2_, Mir); 29.06 (CH_2_, Mir); 28.99 (CH_2_, Mir); 25.50 (CH_2_, Mir); 22.32 (CH_2_, Mir); 13.02 (CH_3_, Mir).

### Determination of Peptide Hydrophobicity with RP-HPLC

To determine peptide hydrophobicity, a Waters Alliance e2695 system with a Waters 2998 PDA Detector was used. All analyses were carried out on a Waters X-Bridge Shield RP-18 column (3.0 × 100 mm, 3.5 μm particle size, 130 Å pore size). The peptides were dissolved in water (0.1% TFA, v/v) up to a concentration of 1 g/L. UV detection at 214 nm was used, and samples (10 μL) were eluted with a linear 20–80% acetonitrile gradient in deionized water over 30 min at 25.0 ± 0.1 °C. The mobile phase flow rate was 0.5 mL/min. Both eluents contained 0.1% (v/v) of TFA. Each peptide sample was analyzed in triplicate. For all analyses, the maximum coefficient of variation was below 0.85%.

### Hemolytic Activity

The hemolysis assay was conducted using a procedure described previously [47]. For this purpose, human red blood cells (RBCs) isolated from K2EDTA Single Donor Human Whole blood (Innovative Research, Inc, MI, USA) were rinsed with Phosphate-buffer saline (PBS, pH 7.4±0.05, composition — KCl 2.7 mM, NaCl 140 mM, phosphate 10mM; AppliChem GmbH, Germany) by centrifugation for 10 min at 800 × g. This process was repeated 3 times and the RBCs were resuspended in PBS. Then, the stock solution of RBCs was added to the serial dilution of lipopeptides on 96-well polystyrene plates to reach a final volume of 100 µL with a 4% concentration of erythrocytes (v/v) and a concentration range of 1–512 µg/mL of tested compounds. The control wells for 0% and 100% hemolysis consisted of RBCs suspended in PBS and 1% of Triton X–100 (Sigma-Aldrich, Germany), respectively. Benzalkonium chloride (Sigma-Aldrich, Germany) was used as reference compound. Subsequently, the plates were incubated for 60 min at 37 °C and then centrifuged at 800 × g for 10 min at 4 °C (Sorvall ST 16R Centrifuge, Thermo Scientific). The supernatant was carefully resuspended to new microtiter plates. The release of hemoglobin was monitored absorbance measurements at 540 nm (Multiskan™ GO Microplate Spectrophotometer, Thermo Scientific). All experiments were conducted in triplicate. HC_50_ was calculated using an ic50.tk tool [48].

### Antimicrobial Assays

#### Cultivation of Microorganisms

The *Acinetobacter baumannii* ATCC BAA-1605, *Enterococcus faecium* ATCC 700221, *Klebsiella aerogenes* ATCC 13048, *Klebsiella pneumoniae* ATCC 700603, *Pseudomonas aeruginosa* ATCC 9027, *Staphylococcus aureus* ATCC 33591, *Candida glabrata* ATCC 15126 strains were acquired from the American Type Culture Collection (ATCC, Manassas, USA). All the strains were stored at −80 °C in Roti-Store cryo vials (Carl Roth GmbH, Germany) and before the tests were transferred into fresh Mueller–Hinton broth (MHB, Becton Dickinson, Poland) for bacteria or RPMI-1640 (Sigma-Aldrich, Germany) for fungi and incubated for 24 h at 37 °C. Then, the cultures were cultured on the Mueller–Hinton agar (BioMaxima, Poland) or Sabouraud dextrose agar (SDA, BioMaxima) plates, respectively, and incubated as just mentioned. For microbiological assays, a minimum of three to five well-isolated colonies of the same morphological type were carefully picked from the agar cultures. These colonies were transferred with sterile loop into fresh microbiological medium and incubated with shaking (100 rpm) at 37 °C until they reached logarithmic phase of growth. Cell densities for all assays were adjusted through spectrophotometric measurements (Multiskan GO Microplate Spectrophotometer, Thermo Fisher Scientific, Finland) at 600 and 530 nm for bacteria and fungi, respectively.

#### Activity Against Planktonic Cultures

The MICs were determined by broth microdilution method according to the Clinical and Laboratory Standard Institute guidelines [49, 50]. For this purpose, initial inoculums of bacteria (5 × 10^5^ CFU/mL) in MHB and yeasts (2 × 10^3^ CFU/mL) in RPMI-1640 with 2% D-glucose were exposed to the ranging concentration of lipopeptides (1–512 μg/mL) and incubated at 37 °C for 18 h and 24 h, respectively. The experiments were conducted on 96-well microtiter polystyrene plates (Kartell, Italy). The growth was assessed visually after incubation and the MIC was assumed as the lowest peptide concentration at which a noticeable growth of microorganisms was inhibited. Benzalkonium chloride (Sigma-Aldrich, Germany) was used as reference compound. All experiments were conducted in triplicate.

### MTS Assay

The immortalized human keratinocyte cell line (HaCaT), obtained from CLS (Cell Line Service, Germany) was cultured in DMEM-high glucose (Dulbecco’s Modified Eagle Medium) supplemented with 10% fetal bovine serum, 2 mM L-glutamine, 100 U/mL penicillin and 100 μg/mL streptomycin (Sigma-Aldrich; Merck KGaA) at 37 °C in a humidified atmosphere with 5% CO_2_. Cells were trypsinized, and the medium was renewed 2–3 times per week. The cytotoxicity of compounds was assessed by colorimetric CellTilter 96^®^ Aqueous One Solution Cell Proliferation Assay (Promega, USA), containing a tetrazolium compound according to the manufacturer’s instructions. In brief, 7 × 10^3^ HaCaT cells per well were plated on 96-well plates and incubated per night to adhere to the cultured dishes. After that time, different concentrations of the tested compounds ranging from 512 to 1 μg/mL were applied to the wells. Every compound was medium-soluble at 37 °C; thus, the non-treated cells were negative control. Serial dilutions of the tested compounds were always prepared before use. Cells were incubated with drugs for 24 h, and then, 20 μL of the CellTiter 96^®^AqueousOne Solution Reagent was added to each well containing treated cells in 100 µl of culture medium. After 4 h of incubation at 37 °C, the absorbance at 490 nm was recorded using a microplate reader (Epoch, BioTek Instruments, USA). The quantity of formazan product as measured by the amount of 490 nm absorbance is directly proportional to the number of living cells in culture [51–53]. Benzalkonium chloride (Sigma-Aldrich, Germany) was used as reference compound. At least three independent experiments with two replicates were conducted for each concentration of a compound, and the IC_50_ values were calculated using GraphPad Prism 8 software.

### Statistical Analysis

GraphPad Prism v. 9.0 and MS Excel software were used to process all data in this work, presented as mean ± standard deviation. Comparisons were performed using Student’s *t*-test. For Hemolysis and Toxicity Curves, two tailed correlation analyses were done. Statistical significance was accepted as a *p*-value of < 0.05.

## Results

### Synthesis

**Scheme 1:**
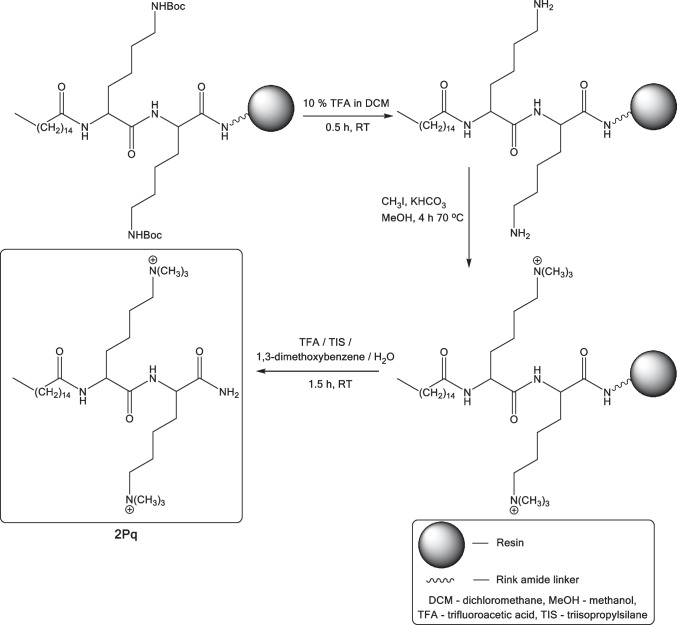
General procedure of the synthesis of QAS derivatives of lipopeptides as presented for the synthesis of **2Pq**

A series of ultrashort cationic lipopeptides containing from 1 to 4 lysine residues and fatty acid substituents: myristic (C_14_), palmitic (C_16_) and stearic (C_18_) were synthesized manually by solid-phase peptide synthesis (SPPS) on the Rink amide resin according to Fmoc/tBu strategy [31, 32]. Resin with rink amide linker was chosen to provide product as C-terminal amide. After purification by reversed-phase high-performance liquid chromatography (RP-HPLC), three series of USCLs were obtained: **1M**-**4M** (M — myristic acid), **1P**-**4P** (P — palmitic acid) and **1S**-**4S** (S — stearic acid). The purity and identity of the compounds were confirmed by LC-MS analyses. The results of mass spectrometry (MS) analyses, sequences, names and adjusted retention times were summarized in Table 1. Another group of USCLs with identical sequences and the modified side chain amino group of lysine residues were also synthesized. In this series, the amino group of the lysine side chain was modified by the attachment of three methyl groups and, in consequence, produced quaternary ammonium salts — quaternized ultrashort cationic lipopeptides (qUSCLs). qUSCLs: **1Mq**-**4Mq**, **1Pq**-**4Pq** and **1Sq**-**4Sq** being analogues of USCLs from the first series with the identical composition of amino acids and fatty acid. The quaternization was performed on the resin during SPPS synthesis by reaction with CH_3_I in the presence of KHCO_3_ in dry methanol at 70 °C. Purification and characterization of qUSCLs were performed analogously to USCLs. Sequences, peptide names and results of MS analyses are summarized in Table 1, while a general scheme of the synthesis of qUSCLs is shown in Scheme 1. Furthermore, relative hydrophobicity was measured by reverse-phase HPLC analyses for peptides. The hydrophobicity of the compounds is correlated with their affinity to hydrophobic stationary phase, i.e. to its retention time. More hydrophobic compounds have higher relative retention times (Table 1).

**Table 1:** Sequences, MS analyses and retention times of the lipopeptides

**Peptide**	**Sequence**	**MS analysis**			**Adjusted retention time (min)**
		***Ion type***	***m/z***^**a**^	***m/z***^**b**^	
**1M**	C_**14**_-K-NH_2_	[M+H]^+^	356.32	356.55	19.55
**2M**	C_**14**_-KK-NH_2_	[M+H]^+^ [M+2H]^2+^	484.42 242.43	484.92 -	15.72
**3M**	C_**14**_-KKK-NH_2_	[M+H]^+^ [M+2H]^2+^	612.52 306.76	612.72 -	13.82
**4M**	C_**14**_-KKKK-NH_2_	[M+H]^+^ [M+2H]^2+^ [M+3H]^3+^	740.60 370.81 185.66	740.75 - -	12.88
**1P**	C_**16**_-K-NH_2_	[M+H]^+^	384.36	384.57	24.11
**2P**	C_**16**_-KK-NH_2_	[M+H]^+^ [M+2H]^2+^	512.45 256.73	512.71 -	19.30
**3P**	C_**16**_-KKK-NH_2_	[M+H]^+^ [M+2H]^2+^ [M+3H]^3+^	640.55 320.78 160.64	640.81 321.00 -	17.01
**4P**	C_**16**_-KKKK-NH_2_	[M+H]^+^ [M+2H]^2+^ [M+3H]^3+^	768.74 384.82 192.66	768.88 - -	15.48
**1S**	C_**18**_-K-NH_2_	[M+H]^+^	412.39	412.62	28.57
**2S**	C_**18**_-KK-NH_2_	[M+H]^+^ [M+2H]^2+^	540.48 270.75	540.62 -	22.38
**3S**	C_**18**_-KKK-NH_2_	[M+H]^+^ [M+2H]^2+^	668.58 334.79	668.77 335.11	19.50
**4S**	C_**18**_-KKKK-NH_2_	[M+H]^+^ [M+2H]^2+^ [M+3H]^3+^	796.67 398.84 266.23	796.84 - -	17.88
**1Mq**	C_**14**_-K(Me_3_)^+^-NH_2_	[M_c_]^+^	398.37	398.60	20.15
**2Mq**	C_**14**_-K(Me_3_)^+^K(Me_3_)^+^-NH_2_	[M_c_]^2+^ [M_c_+FA]^+^	284.76 614.52	285.04 614.70	16.32
**3Mq**	C_**14**_-K(Me_3_)^+^K(Me_3_)^+^K(Me_3_)^+^-NH_2_	[M_c_]^3+^ [M_c_+2FA]^+^	246.96 830.67	- 830.47	14.27
**4Mq**	C_**14**_-K(Me_3_)^+^K(Me_3_)^+^K(Me_3_)^+^K(Me_3_)^+^-NH_2_	[M_c_]^4+^ [M_c_+2FA]^2+^ [M_c_+3FA]^+^	227.96 500.91 1046.82	- 501.32 1046.79	13.13
**1Pq**	C_**16**_-K(Me_3_)^+^-NH_2_	[M_c_]^+^	426.41	426.64	24.39
**2Pq**	C_**16**_-K(Me_3_)^+^K(Me_3_)^+^-NH_2_	[M_c_]^2+^ [M_c_+FA]^+^	298.78 624.55	299.24 642.79	19.77
**3Pq**	C_**16**_-K(Me_3_)^+^K(Me_3_)^+^K(Me_3_)^+^-NH_2_	[M_c_]^3+^ [M_c_+2FA]^+^	256.23 858.70	- 858.76	17.05
**4Pq**	C_**16**_-K(Me_3_)^+^K(Me_3_)^+^K(Me_3_)^+^K(Me_3_)^+^-NH_2_	[M_c_]^4+^ [M_c_+2FA]^2+^ [M_c_+3FA]^+^	234.96 514.92 1074.85	- 515.25 1074.87	15.47
**1Sq**	C_**18**_-K(Me_3_)^+^-NH_2_	[M_c_]^+^	454.44	454.63	29.60
**2Sq**	C_**18**_-K(Me_3_)^+^K(Me_3_)^+^-NH_2_	[M_c_]^2+^ [M_c_+FA]^+^	312.79 670.58	312.97 670.86	23.41
**3Sq**	C_**18**_-K(Me_3_)^+^K(Me_3_)^+^K(Me_3_)^+^-NH_2_	[M_c_]^3+^ [M_c_+2FA]^+^	265.58 886.73	- 886.80	20.23
**4Sq**	C_**18**_-K(Me_3_)^+^K(Me_3_)^+^K(Me_3_)^+^K(Me_3_)^+^-NH_2_	[M_c_]^4+^ [M_c_+2FA]^2+^ [M_c_+3FA]^+^	241.97 528.94 1102.88	- 529.36 1102.74	18.49

^**a**^calculated mass to charge ratio

^**b**^measured mass to charge ratio

**Fig. 2:**
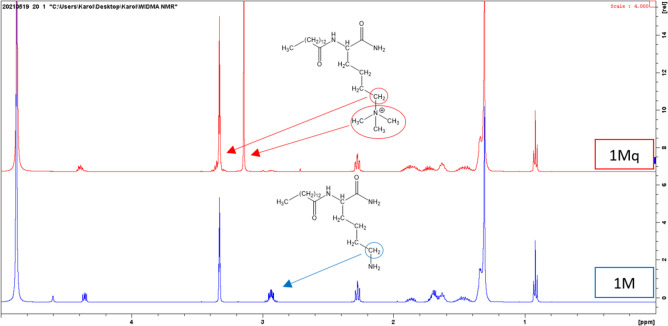
Comparison of ^1^H NMR spectra of lipopeptide **1M** (blue, lower) and the quaternized lipopeptide **1Mq** (red, upper)

To confirm the structure of the products, NMR (^1^H, ^13^C, COSY and HSQC) spectra were recorded for one USCL — **1M** and one qUSCL — **1Mq** counterpart. **1M** and **1Mq** compounds were selected for NMR analyses due to the fact that other USCLs are their homologues with different length of fatty acid residues and different number of lysine residues in sequence. Signals at NMR spectra of multiple lysine residues, which are structurally and magnetically very similar, could overlap each other making spectra unclear and impossible interpret. For that reason, we have chosen the least complex compounds to confirm that the method of quaternization provides expected products. The results of NMR analyses were summarized in the Materials and methods section and Figs. S1–S8 (Supplementary information) presenting copies of NMR spectra. Briefly, despite the finding that ^1^H and ^13^C spectra of **1M** and **1Mq** are very similar to each other, some significant differences must be highlighted. For instance, the presence of a singlet at 3.15 ppm with the integration of 9 protons in the ^1^H NMR spectrum of **1Mq** confirms the attachment of the three methyl groups to the *N*^*ε*^-amino group of lysine. Furthermore, a comparison of proton chemical shift attached to ε carbon atom provides further evidence of amine group modification. In the spectrum of **1M**, the chemical shift of those protons appears at 2.93 while that of **1Mq** at 3.35 ppm. Comparison of ^1^H NMR spectra of **1M** and **1Mq** is presented in Fig. 2.

### Antimicrobial Activity

**Fig. 3:**
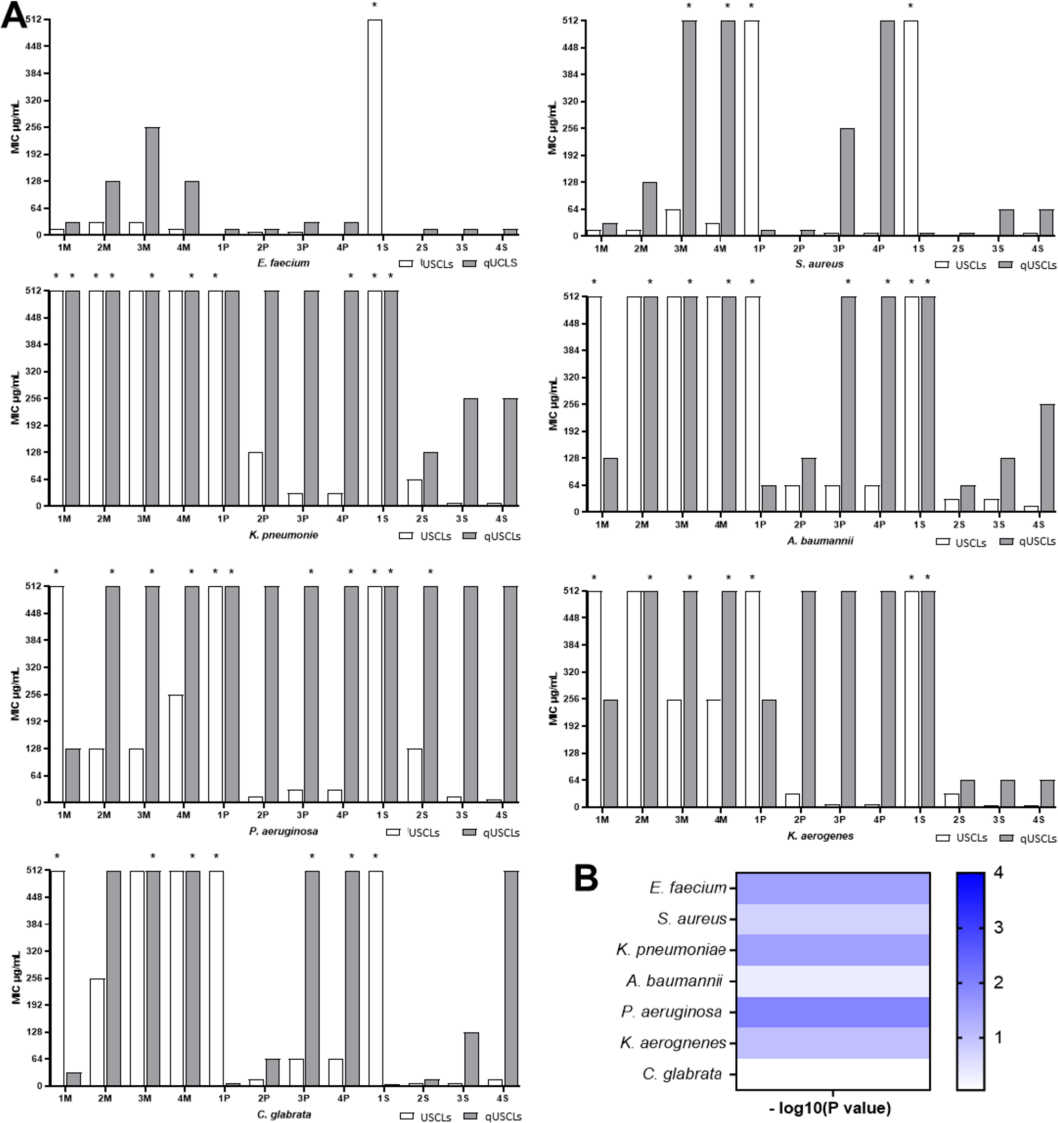
Minimum inhibitory Concentrations (MICs, µg/mL) of test compounds against reference strains of microorganism. **A** Data present MIC values of three independent experimental biological replicates. * Means that MIC > 512 µg/mL. **B** Heatmap presents -log10 (*P*-value) of two-tailed grouped analysis between activity of USCLs vs. qUSCLs of each strain individually

All the synthesized USCLs and qUSCLs were evaluated for their antimicrobial activity against bacteria and fungi. The minimum inhibitory concentration (MIC) of the compounds was determined against bacteria from the ESKAPE group and one fungi strain, *Candida glabrata* ATCC 15126. The group of ESKAPE pathogens comprises clinically important Gram-negative and Gram-positive bacteria with enhanced multidrug resistance and high virulence. ESKAPE bacteria are the following: *Acinetobacter baumannii ATCC BAA 1605*, *Enterococcus faecium ATCC 700221*, *Klebsiella aerogenes* ATCC 13048 (formerly *Enterobacter aerogenes*), *Klebsiella pneumoniae* ATCC 700603, *Pseudomonas aeruginosa* ATCC 9027 and *Staphylococcus aureus* ATCC 33591. The results of MIC determination are summarized in Fig. 3 and Table S1 (Supplementary information).

It can be deduced that Gram-positive strains (*E. faecium* ATCC 700221 and *S. aureus* ATCC 33591) were more susceptible to lipopeptides than the remaining strains. Some of the compounds were inactive to pathogens but majority have their MICs below 512 μg/mL. In general, quaternized lipopeptides were more likely to be inactive according to their mean MIC value and number of inactive compounds wit MIC above 512 μg/mL. Statistical analysis of MIC results for comparison of –log_10_(*P*-value) of two-tailed grouped analysis between activity of USCLs vs. qUCLs of each strain individually was performed and presented as heatmap where the higher value represents the more significant difference between these two groups of compounds (B, Fig. 3). Furthermore, the antimicrobial activity can be affected by net charge and the type of fatty acid. To evaluate the physico-chemical characteristics of the compounds, adjusted retention time (t’R; RP-HPLC) was used as a numeric parameter associated with peptide hydrophobicity. Log_2_MIC values were plotted against t’R to learn how lipophilicity affects antimicrobial activity (Supplementary information; Figs. S12–S24).

### Hemolytic Activity and Cytotoxicity

**Fig. 4:**
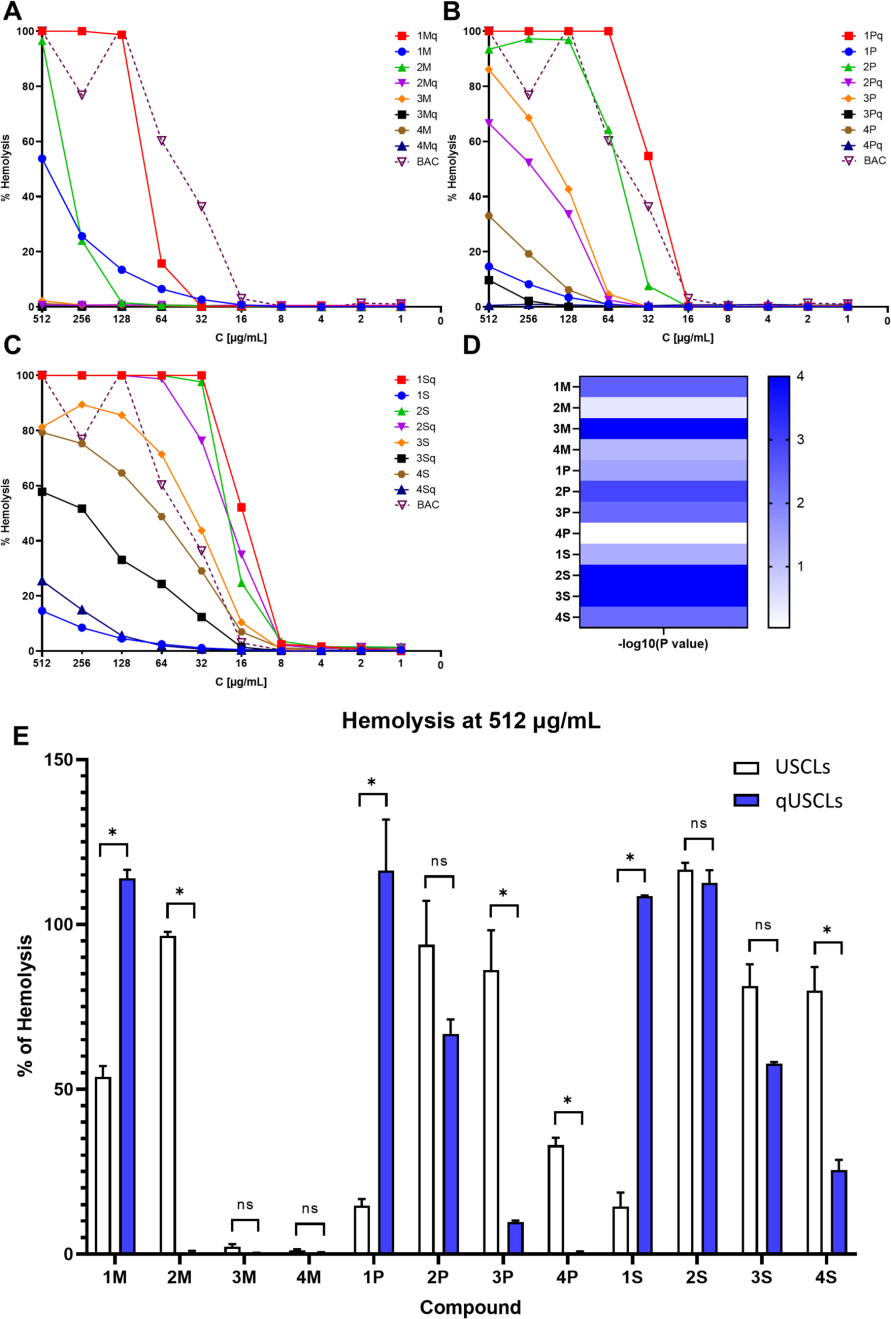
Hemolytic effect of tested USCLs and qUSCLs. Upper images **A**–**C**: Dose-response curves for **A** lipopeptides with C_14_ (M); **B** lipopeptides with C_16_ (P); **C** lipopeptides with C_18_ (S). Benzalkonium chloride (BAC) was used as a reference compound. **D** Heatmap presents -log_10_(*P*-value) of correlation analysis between activity of USCLs vs. qUSCLs. The higher value is, the more significant difference was observed. Lower image (**E**) illustrates the hemolysis at 512 µg/mL. The data represent average values of three independent experimental biological replicates. Error bars depict SE. * *P* < 0.05 was considered significant

**Fig. 5:**
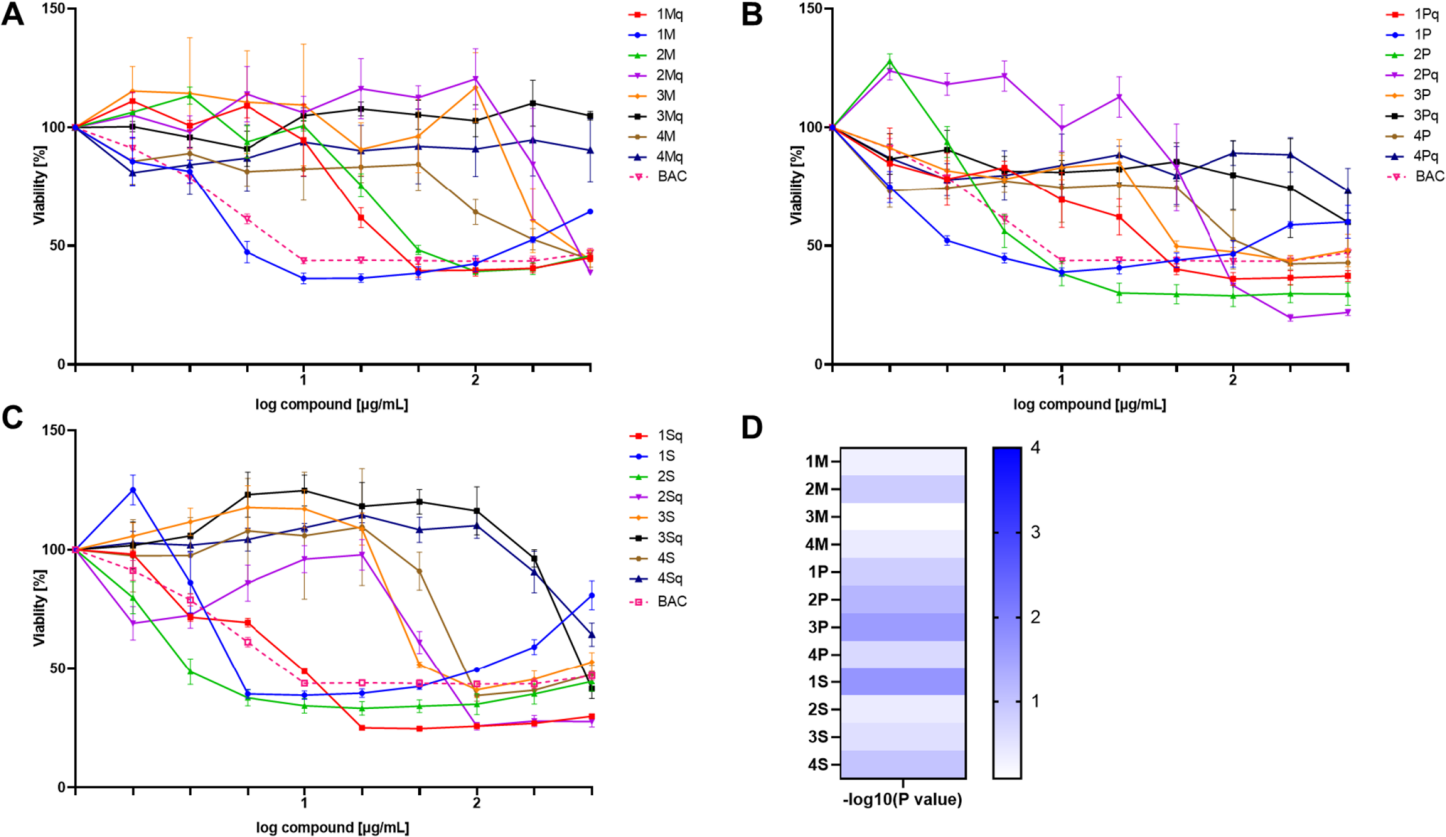
Effect of tested USCLs and qUSCLs on viability of HaCaT. Images **A–C**: Dose-response curves for **A** lipopeptides with C_14_ (M); **B** lipopeptides with C_16_ (P); **C** lipopeptides with C_18_ (S). Benzalkonium chloride (BAC) was used as a reference compound. **D** Heatmap presents -log_10_(*P*-value) of correlation analysis between activity of USCLs vs. qUSCLs. The higher value is, the more significant difference was observed

Hemolytic activity against human red blood cells and cytotoxicity against the human keratinocytes cell line (HaCaT) were determined. Results were summarized in Figs. 4 and 5. Hemolysis results were presented as plots of % of hemolysis of RBCs vs. concentration. Peptides were grouped in three plots depending on type of fatty acid — A — myristic, B — palmitic and C — stearic (Fig. 4). Moreover USCLs vs. qUSCLs were compared in whole range of concentrations and plot of -log_10_(*P-* value) in pairs of lipopeptide and quaternized analogue were prepared (D, Fig. 4), also hemolysis at 512 μg/mL in such pairs was analyzed (E, Fig. 4). Furthermore, statistically significant differences (*P* < 0.05 were denoted at concentration 512 μg/mL in pairs of **1M** vs. **1Mq**,** 2M** vs. **2Mq**, **1P** vs. **1Pq, 3P** vs. **3Pq, 4P** vs. **4Pq**, **1S** vs. **1Sq** and **4S** vs. **4Sq**). When whole concentration range was taken into account, significant differences were observed for almost all pairs of USCLs and qUSCLs except **2M**/**2Mq**, **4M**/**4Mq** and **4P**/**4Pq** with -log_10_(*P-*value) above 1.15 which corresponds to *p* < 0.05 (C, Fig. 4). Similar comparison was made for HaCaT cell line. Plots of % of cell viability *vs*. log(concentration) were prepared and grouped in three plots depending on type of fatty acid — A — myristic, B — palmitic and C — stearic (Fig. 5). In most cases, results suggest that almost all compounds are less cytotoxic than BAC with some exceptions where similar profiles were obtained (**1M**, **1P**, **2P**, **1S**, **1Sq**, **2S**). Moreover, USCLs vs. qUSCLs were compared in whole concentration range of and -log_10_(*P-*value) in pairs of lipopeptide and quaternized analogue were calculated (D, Fig. 5). Most pronounce differences according to statistical analysis were observed in pairs: **1S**/**1Sq** and **3P**/**3Pq**.

### Selectivity Indexes

Half maximal hemolytic concentrations (HC_50_) and half maximal inhibitory concentrations (IC_50_) were calculated for all compounds. In addition, to estimate their potency, selectivity indexes (SI) were calculated for all the strains by dividing the HC_50_ by MIC and IC_50_ by MIC for erythrocytes and HaCaT cell lines respectively. The results are shown in Tables 2 and 3. SI above 1 represents compounds with antimicrobial activity higher than its toxic effect both towards red blood cells and human keratinocytes. In several cases due to lack of antimicrobial activity of compound for specific strain with MIC >512 µg/mL, SI tends to zero. This fact was denoted in Tables 2 and 3 as “-” mark.

**Table 2:** Hemolytic activity (HC_50_ [µg/mL]) and selectivity indexes

**Peptide**	**HC**_**50**_	**Selectivity index (SI)**						
		***E. faecium***	***S. aureus***	***K. pneumoniae***	***A. baumannii***	***P. aeruginosa***	***K. aerogenes***	***C. glabrata***
**1M**	477.61 ± 28.02	**29.85**	**29.85**	-	-	-	-	-
**2M**	345.21 ± 12.80	**10.79**	**21.58**	-	0.67	2.70	0.67	1.35
**3M**	>512	**>16.00**	>8.00	-	>1.00	>4.00	>2.00	>1.00
**4M**	>512	**>32.00**	**>16.00**	-	>1.00	>2.00	>2.00	>1.00
**1P**	>512	**>128.00**	-	-	-	-	-	-
**2P**	55.21 ± 1.69	**6.90**	**13.80**	0.43	0.86	**3.45**	**1.73**	**3.45**
**3P**	135.46 ± 8.69	**16.93**	**16.93**	**4.23**	2.12	**4.23**	**16.93**	2.12
**4P**	>512	**>128.00**	**>64.00**	**>16.00**	>8.00	**>16.00**	**>64.00**	>8.00
**1S**	>512	-	-	-	-	-	-	-
**2S**	21.88 ± 0.68	**5.47**	**10.94**	0.34	**0.68**	0.17	**0.68**	**2.74**
**3S**	32.11 ± 1.54	**8.03**	**8.03**	**4.01**	**1.00**	**2.01**	**8.03**	**4.01**
**4S**	48.44 ± 4.12	**12.11**	**6.06**	**6.06**	**3.03**	**6.06**	**12.11**	**3.03**
**1Mq**	88.25 ± 1.57	**2.76**	**2.76**	-	0.69	0.69	0.34	**2.76**
**2Mq**	>512	>4.00	>4.00	-	-	-	-	>1.00
**3Mq**	>512	>2.00	-	-	-	-	-	
**4Mq**	>512	>4.00	-	-	-	-	-	
**1Pq**	32.21 ± 1.97	**2.01**	**2.01**	0.06	0.50	-	0.13	**4.03**
**2Pq**	133.32 ± 6.27	**8.33**	**8.33**	0.26	1.04	0.26	0.26	2.08
**3Pq**	>512	**>16.00**	>2.00	-	-	-	-	-
**4Pq**	>512	**>16.00**	>1.00	-	-	-	-	-
**1Sq**	16.13 ± 9.88	**4.03**	**2.02**	-	-	-	-	**4.03**
**2Sq**	22.42 ± 0.98	**1.40**	**2.80**	0.18	0.35	-	0.35	**1.40**
**3Sq**	385.50 ± 71.51	**24.09**	6.02	1.51	3.01	0.75	6.02	3.01
**4Sq**	>512	**>32.00**	>8.00	2.00	>2.00	>1.00	>8.00	>1.00
**BAC**	37.98 ± 3.79	9.50	9.50	2.37	2.37	1.19	2.37	9.50

*BAC* benzalkonium chloride

*Bolded values refer to compounds with MIC ≤ 32 µg/mL

**Table 3:** Cytotoxicity (IC_50_ [µg/mL]) and selectivity indexes

**Peptide**	**IC**_**50**_	**Selectivity index (SI)**						
		***E. faecium***	***S. aureus***	***K. pneumoniae***	***A. baumannii***	***P. aeruginosa***	***K. aerogenes***	***C. glabrata***
**1M**	4.33 ± 1.12	**0.27**	**0.27**	-	-	-	-	-
**2M**	32.49 ± 4.84	**1.02**	**2.03**	-	0.06	0.25	0.06	0.13
**3M**	237.60 ± 31.18	**7.43**	3.71	-	0.46	1.86	0.93	0.46
**4M**	252.20 ± 36.68	**15.76**	**7.88**	-	0.49	0.99	0.99	0.49
**1P**	1.74 ± 0.49	**0.44**	-	-	-	-	-	-
**2P**	10.27 ± 1.59	**1.28**	**2.57**	0.08	0.16	**0.64**	**0.32**	**0.64**
**3P**	45.40 ± 20.45	**5.68**	**5.68**	**1.42**	0.71	**1.42**	**5.68**	0.71
**4P**	126.80 ± 27.26	**31.70**	**15.85**	**3.96**	1.98	**3.96**	**15.85**	1.98
**1S**	8.34 ± 1.07	-	-	-	-	-	-	-
**2S**	4.33 ± 0.79	**1.08**	**2.17**	0.07	**0.14**	0.03	**0.14**	**0.54**
**3S**	88.51 ± 4.72	**22.13**	**22.13**	**11.06**	**2.77**	**5.53**	**22.12**	**11.06**
**4S**	155.34 ± 11.20	**38.84**	**19.42**	**19.42**	**9.71**	**19.42**	**38.83**	**9.71**
**1Mq**	24.81 ± 3.88	**0.78**	**0.78**	-	0.19	0.19	0.10	**0.78**
**2Mq**	258.90 ± 35.07	2.02	2.02	-	-	-	-	0.51
**3Mq**	>512	2.00	-	-	-	-	-	-
**4Mq**	>512	>4.00	-	-	-	-		
**1Pq**	19.25 ± 9.34	**1.20**	**1.20**	0.04	0.30	-	0.08	**2.41**
**2Pq**	99.62 ± 9.65	**6.23**	**6.23**	0.19	0.78	0.19	0.19	1.56
**3Pq**	>512	**>16.00**	>2.00	-	-	-	-	
**4Pq**	>512	**>16.00**	>1.00	-	-	-	-	
**1Sq**	14.13 ± 0.39	**3.53**	**1.77**	-	-	-	-	**3.53**
**2Sq**	78.57 ± 5.49	**4.91**	**9.82**	0.61	1.23	-	1.23	**4.91**
**3Sq**	474.64 ± 29.06	**29.67**	7.42	1.85	3.71	0.93	7.42	3.71
**4Sq**	>512	**>32.00**	>8.00	>2.00	>2.00	>1.00	>8.00	>1.00
**BAC**	11.70 ± 0.76	2.88	2.88	0.72	0.72	0.36	0.72	2.88

*BAC* benzalkonium chloride

*Bolded values refer to compounds with MIC ≤ 32 µg/mL

Furthermore, biological activity parameters — HC_50_, IC_50_, SIs — were plotted against adjusted retention times (t’R) to find out how the hydrophobicity affects them. Fig. S11 include HC_50_, Fig. S26 — IC_50_, Figs. S27–S40 — SIs based on MICs and HC_50_, and Figs. S41–S54 — SIs based on MICs and IC_50_.

## Discussion

The purpose of this study was to evaluate the influence of quaternization of the amino group of the side chain of lysine on the antimicrobial, hemolytic and cytotoxic activities of USCLs. To achieve this, two series of ultrashort cationic lipopeptides containing lysine residues and fatty acid groups and their quaternary ammonium analogues were designed. In this study, three types of fatty acids were used — myristic (C_14_), palmitic (C_16_) and stearic (C_18_) which were coupled with peptides composed of 1–4 lysine residues. Another series of compounds consisted of quaternary ammonium salts, analogues of the synthesized USCLs with modified side chain amino groups by attachment of three methyl groups. Our goal was to introduce quaternization reaction as one of the steps of solid-phase peptide synthesis. We believe that this approach is the most effective and less time- and cost-consuming than derivatization in the solution of USCLs or Fmoc-L-Lys-OH. In the case of USCLs quaternization in solution, it is necessary to isolate modified qUSCLs, but it might be problematic (chromatographic purification of a complex mixture). On the other hand, removal of reagents from the resin with attached qUSCLs is quite straightforward including simple washing and draining within the standard SPPS protocol. Another possibility is either modification of Fmoc-L-Lys-OH (quaternization in solution) or the use of commercially available Fmoc-L-Lys(Me_3_)^+^-OH in SPPS. Quaternization of Fmoc-L-Lys-OH is possible, but it seems to be rather unfavorable due to additional steps (reaction, isolation, identification and purification). Finally, the main drawback of the usage of Fmoc-L-Lys(Me_3_)^+^-OH is its expensiveness — the costs of Fmoc-L-Lys(Boc)-OH or Fmoc-L-Lys-OH and reagents required to quaternization are many times lower. A general procedure for the synthesis of qUSCLs is presented in Scheme 1. The main steps of the synthesis included the elongation of the peptide chain up to its final sequence; removal of Boc protecting groups from *N*^*ε*^-amino groups of lysine by 10% TFA in DCM, resin washing to remove by-products and the reaction with CH_3_I in the presence of KHCO_3_ in methanol to obtain the desired qUSCLs (Scheme 1). However, before the syntheses with peptides anchored to the resin, we optimized reaction conditions with unprotected lysine as a model compound. In the literature, there are two approaches to the methylation of lysine: one with CH_3_I [33, 34] and the other with dimethylsulfate as the methylating agent [35]. The reactions were carried out at room temperature according to the literature procedure, but no product formation was noticed as shown by LC-MS analyses. Next, we performed the reactions at an elevated temperature (60 °C) and this led to the formation of the desired product, i.e. *N*^*α*^*,N*^*ε*^-hexamethyl-L-lysine, but only when methyl iodide was used. A next reaction with methyl iodide was performed for on-resin modification of the peptides, with slight optimization by raising the temperature from 60 to 70 °C to ensure effective quaternization of all lysine residues. This modification was crucial for peptides containing four lysine residues, where reaction at 60 °C gave after 4 h a mixture of products of incomplete methylation. Finally, the peptides were cleaved from the resin, and analogously to USCLs, purified by RP-HPLC and characterized by LC-MS analyses (Table 1). In addition, two compounds, one USCL and qUSCL, were analyzed by NMR (^1^H, ^13^C, COSY and HSQC). According to our knowledge, this is the first study presenting on-resin peptide quaternization. Developed method can be applied for quaternization of other peptides, including antimicrobial ones. Evaluation of hydrophobicity of USCLs and qUSCLs was conducted by analysis of retention times in reverse-phase HPLC (Table 1). Briefly, the hydrophobicity of the compounds is correlated, i.e. their affinity to the hydrophobic stationary phase thus to its retention time. We noticed a correlation between adjusted retention times (t’R) and the number of lysine residues in a series of different fatty acids. Retention times of USCLs and qUSCLs were exponentially correlated with the number of amino acids. In general, retention time and hydrophobicity fall with an increase in the number of lysine residues (Fig. S9, Supplementary information). Moreover, the difference between adjusted retention times of qUSCLs and the corresponding USCLs (Δt'R) is correlated with the number of amino acid residues, as a quadratic function (Fig. S10, Supplementary information). In general, quaternization is affected in more lipophilic compounds (Δt'R = t’R_qUSCL_ – t’R_USCL_), as can be seen, with the increasing number of amino acid residues, the difference is declining. Interestingly, analogous Δt’Rs between the series increase as follows C_16_ < C_14_ < C_18_.

The synthesized USCLs and their qUSCLs analogues 12 compounds each were evaluated for their antibacterial, antifungal, hemolytic and cytotoxic activities. After a comparison of MIC values of USCLs and qUSCLs, a general statement can be formulated, that the quaternization of lysine residues does not stimulate antibacterial and antifungal activities. This observation is consistent with previous findings of Infante et al. Study on *N*^*α*^-lauroyl-L-lysine derivatives revealed that lysine quaternization (e.g. LLM – *N*^*α*^-Lauroyl-L-Lysine methyl ester vs. LLMq – *N*^*α*^-Lauroyl-*N*^*ε*^*N*^*ε*^*N*^*ε*^-trimethyl-L-Lysine methyl ester) results in decreased antimicrobial activity against both, Gram-positive and Gram-negative strains [36]. In some cases, comparable MICs were noticed and in one case, for peptides with stearic acid and one lysine (**1S** and **1Sq**) significant differences in activities were found. The quaternized analogue (**1Sq**) proved to be very effective against *S. aureus*, *E. faecium* and *C. glabrata* (MIC 4-8 µg/mL), while USCL (**1S**) did not show any activity (MIC >512 µg/ml). In general, the most susceptible microorganisms were Gram-positive bacteria — *S. aureus* and *E. faecium*. For *S. aureus*, the highest activity was noted for lipopeptides **2P**, **3P**, **4P**, **2S**, **3S**, and **4S** (MIC 2-8 µg/mL). Interestingly, significant differences in activity between some analogues could be distinguished, i.e. with peptides **3P** and **4P** (MIC 8 µg/mL) and quaternized USCLs **3Pq** and **4Pq** (MIC 256 and 512 µg/mL) a dramatic drop in activity following quaternization was seen. All USCLs exhibited high and moderate activity against *E. faecium* with MIC ranging between 4 and 32 µg/mL with one exception, **1S,** where no activity could be seen over the tested concentration range. The most distinct activity among qUSCLs was found for **1Pq**-**4Pq** and **2Sq-4Sq** (MIC 16-32 µg/mL), with an exceptional one for **1Sq** (MIC 4 µg/mL). Out of all the compounds only USCLs **3P**, **4P**, **3S** and **4S** with MIC 32, 32, 8 and 8 µg/mL respectively exhibited activity against *K. pneumoniae*. Similar results were obtained for *P. aeruginosa*, *A. baumannii* and *K. aerogenes*. Only lipopeptides **2P, 3S** and **4S** exhibited strong activity against *P. aeruginosa* with MIC of 16, 16 and 8 µg/mL, respectively. Interestingly, **1P** failed to show any activity against *A. baumannii,* but its quaternized analogue (**1Pq**) inhibited the growth of bacteria at 64 µg/mL. An interesting situation presented *C. glabrata*. Among USCLs, the highest activity displayed **2S**, **3S** and **2P**, the peptides containing 2 and 3 lysine residues. Again, among qUSCLs, the most active compounds were those with single lysine — **1Mq**, **1Pq** and **1Sq** with MIC 32, 8 and 4 µg/mL respectively. Moreover, **1S** proved to be inactive over the tested concentration range, but its quaternized analogue **1Sq** was very active against *C. glabrata*. Highest statistically significant differences between USCLs and qUSCLs in antimicrobial activity were observed for *P. aeruginosa > K. pneumonia, E. faecium* > *K. aerogenes > S. aureus.* All results were summarized in Fig. 2 and Table S2 (Supplementary information). In this study, BAC was used as reference compound, generally most of USCLS and qUSCLs were less active against all strains of microorganisms, because BAC exhibited high activity towards both Gram-positive and Gram-negative strains while lipopeptides were more potent against Gram-positive bacteria. However, in some cases, similar or superior activity was noted. For example, **1Sq** identical activity against *C. glabrata* (MIC 4 µg/mL) and *E. faecium* (MIC 4 µg/mL) or **2S** was more active against *S. aureus (*MIC 2 vs. 4 µg/mL). Furthermore, USCLs 3S and 4S were more active against *K. pneumonia, K. aerogenes, P. aeruginosa and A. baumannii.*

The determined MIC values (between 2 and 512 μg/mL) were plotted against t’R (Supplementary information, Figs. S12–S25). In some cases, no clear correlations were found, but usually, there were moderate to strong (*R*^2^ between 0.3751 and 0.9071). The linear correlation between log_2_MIC and t’R shows that an increase in lipophilicity of USCLs and qUSCLs is beneficial to their antimicrobial activity. Interestingly, the quadratic function depicting the relation between the antibacterial activity of USCLs against *P. aeruginosa* and the adjusted retention time indicates that there is an optimum hydrophobicity of this group of compounds (minimum of the function). Occurrences of such minimums were reported in the literature [5, 7, 30]. Moreover, this suggests that in the case of other microorganisms used in this study, more hydrophobic USCLs and qUSCLs (e.g. containing arachidic acid residue — C_20_) than those already synthesized could have lower MICs. However, primary studies on methyl esters of short cationic lipopeptides indicate that further elongation of fatty acid chain (i.e. C_20_) doesn’t necessarily enhance antimicrobial potency [37]. Nevertheless, it was observed in other studies on lysine-rich USCLs with C-terminal amide that the highest antimicrobial activity was for those lipopeptides with the longest fatty acid chain (hexadecanoic acid, C_16_) [4]. Indeed, our results confirmed that extended hydrocarbon chain (C_18_) can result in USCLs with lower MIC that encourages to use fatty acid residues with higher number of carbon atoms in further studies.

Analyses of biological activities and retention behavior showed that compounds **1M**, **1P** and **1S** are surprisingly, the least effective antimicrobials among USCLs. Oppositely, analogues qUSCLs are usually those with the highest antimicrobial activity in each group (**Mq**, **Pq**, **Sq**). Hypothetically, compounds **1M**, **1P**, and **1S** are prone to aggregate and precipitate in the medium and therefore their soluble fraction is substantially reduced, as seen in the biological activity. Presumably, those phenomena can also be applied to other conducted experiments and are likely to impact the results. As can be seen in Fig. S11 (HC_50_ vs. t’R), **1M**, **1P** and **1S** did not follow the trend. Different results and conclusions in the case of quaternized analogues (**1Mq**, **1Pq**, **1Sq**) presumably are due to their enhanced solubility. Examples are confirming the enhanced solubility in water of the quaternary ammonium salts vs. analogous amines, e.g. glycine is noticeably less soluble in water than betaine (*N,N,N*-trimethylglycine), 249 g/L vs. 611 g/L, respectively [38, 39]. Nevertheless, this issue needs further research. High water solubility can facilitate drug bioavailability. It can be presumed that improved solubility of quaternized compounds in comparison to their parent molecules can be beneficial to their applicability, especially in pharmacy. To evaluate the toxicity of the compounds, erythrocytes and a human keratinocytes cell line were used. For lipopeptides **1Pq**, **1Sq**, **1S**, **2Sq**, **1Mq**, **2P**, **3S**, **4S** similar to BAC profiles of hemolysis were obtained. More insight brings analysis of calculated HC_50_ values. HC50 for BAC was 37.98 μg/mL and only **2S**, **1Sq** and **2Sq** lipopeptides were more hemolytic than BAC. For **1Pq**, **3S** and **4S** similar to BAC HC_50_ values were obtained, all other USCLs and qUSCLs were less hemolytic (Table 2 and Fig. 4A–C). Comparison of hemolytic activity of USCLs and qUSCLs showed that hemolysis of **1M** vs. **1Mq**, **2M** vs. **2Mq**, **1P** vs. **1Pq**, **3P** vs. **3Pq**, **4P** vs. **4Pq**, **1S** vs. **1Sq** and **4S** vs. **4Sq** is statistically different (*P* < 0.05) when analyzing hemolysis at concentration 512 μg/mL (Fig. 4D). Furthermore, when whole concentration range was taken in to account, significant differences were observed for almost all pairs of USCLs and qUSCLs except **2M**/**2Mq**, **4M**/**4Mq** and **4P**/**4Pq** (C, Fig. 4). Toxicity against human cell line (HaCaT) was also evaluated. Analysis of plots of % of cell viability vs. log(concentration) (Fig. 5). In most cases, results suggest that almost all compounds are less cytotoxic than BAC with some exceptions where similar profiles were obtained (**1M**, **1P**, **2P**, **1S**, **1Sq**, **2S**). Moreover, USCLs vs. qUSCLs were compared in whole range of concentrations. According to -log10(*P*-value), for pairs of lipopeptide and quaternized analogue, most pronounce differences in cytotoxicity was observed for pairs: **1S**/**1Sq** and **3P**/**3Pq** (D, Fig. 5). Furthermore, IC_50_ values suggest that except **1M**, **1P**, **2P**, **1S**, **1Sq**, **2S** all USCLs and qUSCLs were less toxic than BAC (IC_50_ 11.7 μg/mL).

We have investigated correlation between hydrophobicity of lipopeptides with hemolytic and cytotoxic activities. Figs. S11 and S26 (Supplementary information) display t’Rs, HC_50_ and IC_50_ values, respectively. It can be deduced that those values are exponentially correlated with peptides hydrophobicity, but those correlations are moderate with R^2^ between 0.5207 and 0.8549 (**1M** was excluded). In general, the toxicity of the lipopeptides elevates with growing lipophilicity (t’R) and these observations are in agreement with our previous findings [7, 40]. It was expected to observe increasing toxicity with growing lipid chain but our results obtained for USCLs with one lysine residue are in contrary to this expectation. Similar observations were made by Pérez et al. [41]. Study on *N*^ε^-acyl lysine methyl esters revealed that hemolytic activity decreases with lipid chain elongation [41]. Based on the determined exponential functions, it can be stated that qUSCLs are less hemolytic and cytotoxic than USCLs, i.e. qUSCLs of hydrophobicity comparable with that of USCLs exhibit higher HC_50_ and IC_50_ values. Similar conclusions were drawn by Fernandez-Reyes et al. [10]. Substitution of lysine residues with *N*^*6*^*,N*^6^*,N*^6^-trimethyl-L-lysine leads to CA(1-7)M(2-9) (CAMEL — cecropin A and a melittin hybrid peptide) analogues of reduced hemolytic activity and improved selectivity to bacterial pathogens and *Leishmania* parasites. Moreover, it was shown that quaternization reduced antimicrobial activity, but in some cases could improve selectivity owing to a concomitant reduction in toxicity [10, 42]. Interestingly, different results were obtained for lysine-based dimeric surfactant. Colomer et al. found that quaternized analog was substantially more hemolytic than parent molecule [22].

More insight into the activity of USCLs and qUSCLs arises from the analysis of selectivity indexes (SI), for both groups hemolytic against red blood cells and cytotoxic against human keratinocytes activities. Furthermore, selectivity indexes were calculated (Tables 2 and 3) and plotted against adjusted retention time (Supplementary information, Figs. S27–S54). It can be concluded that in a few cases, there is a moderate correlation between SI (HC_50_) and t’R, namely *E. faecium* and USCLs (*R*^2^ = 0.5492), *K. pneumoniae* and USCLs (*R*^2^ = 0.5355), *K. pneumoniae* and qUSCLs (*R*^2^ = 0.5925), *C. glabrata* and USCLs (*R*^2^ = 0.9286). In general, it can be deduced that except *C. glabrata*, the growing hydrophobicity effect is associated with a suppressed selectivity. Similar conclusions can be made in terms of IC_50_-related SI — increasing t’R is accompanied by reduced SI. In the case of selectivity to pathogens over human keratinocytes, it was helpful to divide compounds into three groups — **M** (C_14_), **P** (C_16_) and **S** (C_18_). This approach enabled to spot a high correlation (*R*^2^ between 0.6160 and 0.9995) in the case of SI of USCLs and all strains, but there are only two examples of such a correlation in quaternized lipopeptides (**Sq** and *E. faecium*, and *S. aureus*).

Importantly, one of hypothesis of this study is elevated selectivity of quaternized lipopeptides in comparison to parent molecules (lipopeptides with free amine groups). Therefore, in order to determine whether or not quaternization improves lipopeptides selectivity, SIs were evaluated. The analysis of SIs (Supplementary information, Tables S2 and S3) indicates that approximately half of the calculated quotients are >1 when as far as IC_50_ is considered, but only a few in the case of HC_50_. This result indicates that quaternized analogues can be more selective than their parent molecules and this finding is in agreement with the literature [10]. It is well known that USCLs and QAS interact with cell membranes. It is postulated that selectivity bases on differences between pathogens’ and mammalian membranes — first one is rich in negatively charged components while the latter in zwitterionic ones. Positively charged surfactants interact with negatively charged membrane components, e.g. phospholipids [43, 44]. Cell lysis is an effect of membrane permeabilization and leakage of cell constituents. On the other hand, mammalian cell membranes consist of mainly zwitterionic lipids (e.g. phosphatidylcholine) and therefore interactions between cationic surfactants and cell membrane are diminished. It is worth noting that another interaction that plays crucial role is H-bonding between amine group (-NH_2_) of surfactant and phospholipids of cell membrane. These interactions occur in case of bacterial membrane as well as mammalian ones. However, despite differences between cell membranes, cytotoxicity of USCLs and QAS is still a significant issue. The study of Fernandez-Reyes et al. [10] on quaternized analogs of CAMEL peptide suggests that enhanced selectivity is a result of reduced number of H-bonds between phospholipids and quaternized analogs in comparison to CAMEL. Similar presumption can be made in case of qUSCLs used in this study.

Enhanced selectivity and decreased toxicity of new antibiotics are desired and therefore a class of qUSCLs can provide candidates for further drug research. In consequence, antimicrobial potency and improved selectivity predestinate qUSCLs to be applied as preservatives, antibiotics, and biomaterials components for prevention of microbial infections.

### Future Perspectives and Limitations of this Study

There are several potential limitations of the present study that could be addresed in the future research. USCLs with stearic acid residue (**S**; the longest one) were most selective even if their hydrophobicity was similar to that of compounds with palmitic (**P**) or myristic acids (**M**). This finding stimulates further investigation of qUSCLs with longer fatty acid residues (e.g. C_20_ — icosanoic acid) and emphasizes the importance of a balance between hydrophilicity and lipophilicity. Our previous studies revealed that branched carboxylic acids can provide USCLs with improved antimicrobial activity and selectivity [7]. Therefore, those acids could be used for synthesis of new qUSCLs with superior biological characteristics. Moreover, usage of different basic amino acid residues, e.g. ornithine or histidine, should be considered. Another aspect that can be extended are biological studies. Here, reference strains of ESKAPE group were used but focus on clinical-relevant strains such as *S. aureus*, should be used in future research. Results on clinical strains can bring new shed of light on antimicrobial activity and some discrepancies from those results obtained with reference strains. Furthermore, evaluation of cytotoxicity can be enriched with other cell lines, e.g. fibroblasts or HeLa [45, 46].

## Conclusions

The increasing microbial drug resistance is one of the major human threats and therefore new drug development is crucial for medicine and pharmacy nowadays. USCLs are strong antimicrobial agents and can be considered drug candidates. This study has been focused on USCLs as potent antimicrobials but with one serious flaw — low selectivity. The study aimed to learn how the quaternization of USCLs affects biological activity. The main findings are the following:

- Antimicrobial activity of USCLs and qUSCLs against ESKAPE pathogens and *C. glabrata* is elevating with increasing hydrophobicity, but qUSCLs are usually poorer antimicrobial agents than their parent molecules. Generally, USCLs and qUSCLs are more active against Gram-positive bacteria (*S. aureus* and *E. faecium*). Among qUSCLs, the most active compounds were those with single lysine — **1Mq**, **1Pq** and **1Sq.** Furthermore, **1Sq** — quaternized lipopeptide with single lysine and steric acid showed superior activity against *C. glabrata* in comparison to its USCLs analogue (**1S**);
- Hemolytic activity and cytotoxicity to HaCaT cell line are increasing with hydrophobicity but quaternized analogs are less toxic than the parent molecules of similar t’R. Furthermore, hemolytic and cytotoxic activities decrease with the increased number of lysine residues, both for USCLs and qUSCLs;
- Quaternization can lead to higher selectivity of lipopeptides, but it is more pronounced in keratinocytes than in erythrocytes. USLCs — **4M**, **4P**, **4S** and qUSCLs **3Sq**, **4Sq** proved to be most selective when compared IC_50_ and MIC for *E. faecium and S. aureus.* Quaternized USCLs **2Sq** proved to be selective and active against *S. aureus* with IS and MIC of 9.8 and 8 μg/mL respectively. Furthermore, **1Sq** and **2Sq** were selective and active against *C. glabrata* with MIC 4 and 16 μg/mL; and IS 3.5 and 4.9 respectively;
- Hypothetically, quaternization can enhance peptides’ solubility and therefore their utility;
- Quaternization of lysine residues in USCLs can be performed on-resin with CH_3_I and KHCO_3_ in methanol as a step of SPPS.

This study demonstrates that quaternization is not a remedy for nonselective lipopeptides, but rather offers a possibility for their improvement.

### Supplementary Information

Below is the link to the electronic supplementary material.

Supplementary file1 (DOCX 12336 KB)

## Data Availability

The data presented in this study are available on request from the corresponding author.
