# Supplementary material for: Quaternary Ammonium Salts of Cationic Lipopeptides with Lysine Residues — Synthesis, Antimicrobial, Hemolytic and Cytotoxic Activities
Source: Probiotics Antimicrob Proteins. 2023 Sep 29;15(6):1465–83. doi: 10.1007/s12602-023-10161-8 (PMC10687119; doi:10.1007/s12602-023-10161-8)
Supplement: Supplementary file 1 — Supplementary file1 (DOCX 12336 KB) [file 12602_2023_10161_MOESM1_ESM.docx]

**Supplementary materials**

Quaternary Ammonium Salts of Cationic Lipopeptides with Lysine Residues — Synthesis, Antimicrobial, Hemolytic and Cytotoxic Activities

Karol Sikora^a*^, Jakub Jędrzejczak^a^, Marta Bauer^a^, Damian Neubauer^a^, Maciej Jaśkiewicz^b^, Magdalena Szaryńska^c^

*^a^ Department of Inorganic Chemistry, Faculty of Pharmacy, Medical University of Gdańsk, Al. Gen. J. Hallera 107,
80-416 Gdańsk, Poland*

*^b^ International Research Agenda 3P- Medicine Laboratory, Dębinki 7, building no. 5, 80-211 Gdańsk, Poland*

*^c^ Department of Histology, Faculty of Medicine, Medical University of Gdańsk, Dębinki 1, 80-211 Gdańsk, Poland*

*^*^ Corresponding author.*

*E-mail address:* [*karol.sikora@gumed.edu.pl*](mailto:karol.sikora@gumed.edu.pl)

***TABLE OF CONTEST***

[1. Copies of NMR spectra for 1M and 1Mq 3](#_Toc144988804)

[2. Retention time 7](#_Toc144988805)

[3. HC_50_ *vs.* hydrophobicity 8](#_Toc144988806)

[4. Antimicrobial activity – MIC values 9](#_Toc144988807)

[5. Antimicrobial activity vs. hydrophobicity 10](#_Toc144988808)

[4.1. E. faecium ATCC 700221, USCLs 10](#_Toc144988809)

[4.2. E. faecium ATCC 700221, qUSCLs 10](#_Toc144988810)

[4.3. S. aureus ATCC 33591, USCLs 11](#_Toc144988811)

[4.4. S. aureus ATCC 33591, qUSCLs 11](#_Toc144988812)

[4.5. K. pneumoniae ATCC 700603, USCLs 11](#_Toc144988813)

[4.6. K. pneumoniae ATCC 700603, qUSCLs 12](#_Toc144988814)

[4.7. A. baumannii ATCC BAA 1605, USCLs 12](#_Toc144988815)

[4.8. A. baumannii ATCC BAA 1605, qUSCLs 12](#_Toc144988816)

[4.9. P. aeruginosa ATCC 9027, USCLs 13](#_Toc144988817)

[4.10. P. aeruginosa ATCC 9027, qUSCLs 13](#_Toc144988818)

[4.11. K. aerogenes ATCC 13048, USCLs 13](#_Toc144988819)

[4.12. K. aerogenes ATCC 13048, qUSCLs 14](#_Toc144988820)

[4.13. C. glabrata ATCC 15126, USCLs 14](#_Toc144988821)

[4.14. C. glabrata ATCC 15126, qUSCLs 14](#_Toc144988822)

[6. IC_50_ *vs.* hydrophobicity 15](#_Toc144988823)

[7. Selectivity indexes (SIs) – MIC and HC_50_ *vs.* t’R 16](#_Toc144988824)

[6.1. MICs of USCLs against E. faecium ATCC 700221 and HC_50_ vs. hydrophobicity 16](#_Toc144988825)

[6.2. MIC of qUSCLs against E. faecium ATCC 700221 and HC_50_ vs. hydrophobicity 16](#_Toc144988826)

[6.3. MIC of USCLs against S. aureus ATCC 33591 and HC_50_ vs. hydrophobicity 17](#_Toc144988827)

[6.4. MIC of qUSCLs against S. aureus ATCC 33591 and HC_50_ vs. hydrophobicity 17](#_Toc144988828)

[6.5. MIC of USCLs against K. pneumoniae ATCC 700603 and HC_50_ vs. hydrophobicity 18](#_Toc144988829)

[6.6. MIC of qUSCLs against K. pneumoniae ATCC 700603 and HC_50_ vs. hydrophobicity 18](#_Toc144988830)

[6.7. MIC of USCLs against A. baumannii ATCC BAA 1605 and HC_50_ vs. hydrophobicity 19](#_Toc144988831)

[6.8. MIC of qUSCLs against A. baumannii ATCC BAA 1605 and HC_50_ vs. hydrophobicity 20](#_Toc144988832)

[6.9. MIC of USCLs against P. aeruginosa ATCC 9027 and HC_50_ vs. hydrophobicity 20](#_Toc144988833)

[6.10. MIC of qUSCLs against P. aeruginosa ATCC 9027 and HC_50_ vs. hydrophobicity 21](#_Toc144988834)

[6.11. MIC of USCLs against K. aerogenes ATCC 13048 and HC_50_ vs. hydrophobicity 21](#_Toc144988835)

[6.12. MIC of qUSCLs against K. aerogenes ATCC 13048 and HC_50_ vs. hydrophobicity 22](#_Toc144988836)

[6.13. MIC of USCLs against C. glabrata ATCC 15126 and HC_50_ vs. hydrophobicity 22](#_Toc144988837)

[6.14. MIC of qUSCLs against C. glabrata ATCC 15126 and HC_50_ vs. hydrophobicity 23](#_Toc144988838)

[8. Selectivity indexes (SIs) – MIC *vs.* IC_50_ 24](#_Toc144988839)

[7.1. MICs of USCLs against E. faecium ATCC 700221 and IC_50_ vs. Hydrophobicity 24](#_Toc144988840)

[7.2. MIC of qUSCLs against E. faecium ATCC 700221 and IC_50_ vs. hydrophobicity 25](#_Toc144988841)

[7.3. MIC of USCLs against S. aureus ATCC 33591 and IC_50_ vs. hydrophobicity 26](#_Toc144988842)

[7.4. MIC of qUSCLs against S. aureus ATCC 33591 and IC_50_ vs. hydrophobicity 27](#_Toc144988843)

[7.5. MIC of USCLs against K. pneumoniae ATCC 700603 and IC_50_ vs. hydrophobicity 28](#_Toc144988844)

[7.6. MIC of qUSCLs against K. pneumoniae ATCC 700603 and IC_50_ vs. hydrophobicity 28](#_Toc144988845)

[7.7. MIC of USCLs against A. baumannii ATCC BAA 1605 and IC_50_ vs. hydrophobicity 29](#_Toc144988846)

[7.8. MIC of qUSCLs against A. baumannii ATCC BAA 1605 and IC_50_ vs. hydrophobicity 29](#_Toc144988847)

[7.9. MIC of USCLs against P. aeruginosa ATCC 9027 and IC_50_ vs. hydrophobicity 30](#_Toc144988848)

[7.10. MIC of qUSCLs against P. aeruginosa ATCC 9027 and IC_50_ vs. hydrophobicity 30](#_Toc144988849)

[7.11. MIC of USCLs against K. aerogenes ATCC 13048 and IC_50_ vs. hydrophobicity 31](#_Toc144988850)

[7.12. MIC of qUSCLs against K. aerogenes ATCC 13048 and IC_50_ vs. hydrophobicity 31](#_Toc144988851)

[7.13. MIC of USCLs against C. glabrata ATCC 15126 and IC_50_ vs. hydrophobicity 32](#_Toc144988852)

[7.14. MIC of qUSCLs against C. glabrata ATCC 15126 and IC_50_ vs. hydrophobicity 32](#_Toc144988853)

[9. Comparison SIs between analogs 33](#_Toc144988854)

# Copies of NMR spectra for 1M and 1Mq

*
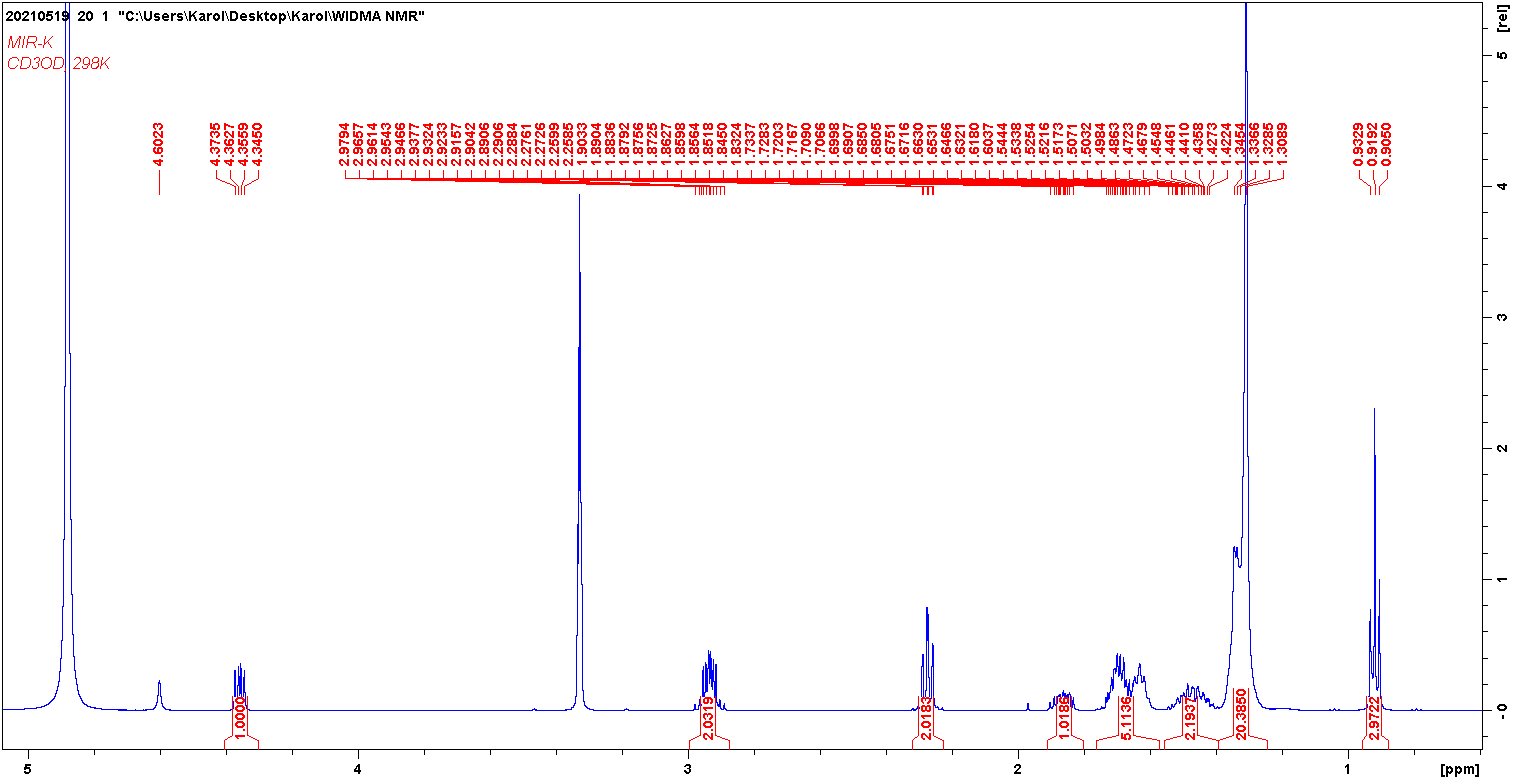
*

**Figure S1.** ^1^H NMR (CD_3_OD, 500 MHz) spectrum of **1Mq**.

*
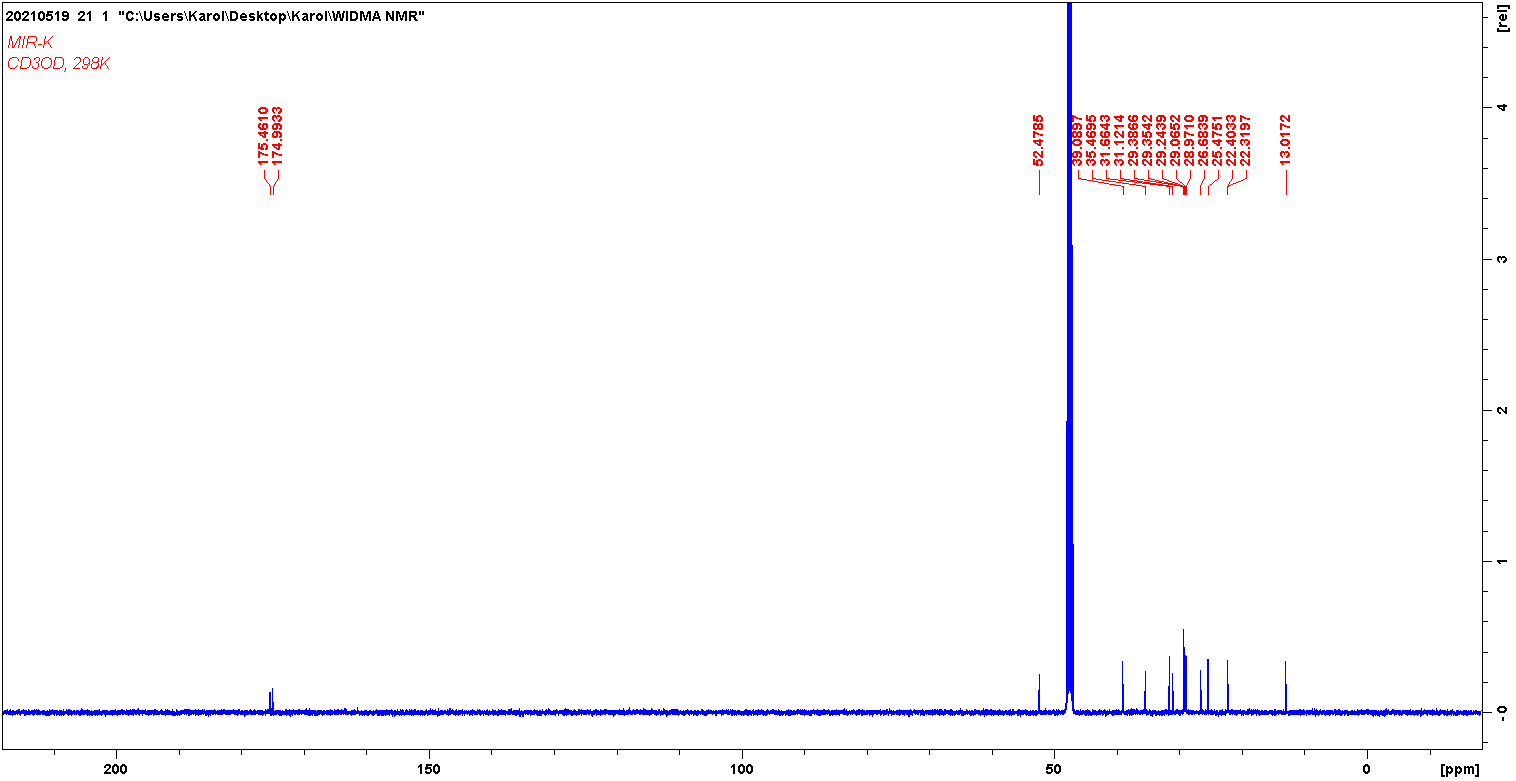
*

**Figure S2.** ^13^C NMR (CD_3_OD, 125 MHz) spectrum of **1Mq**.

*
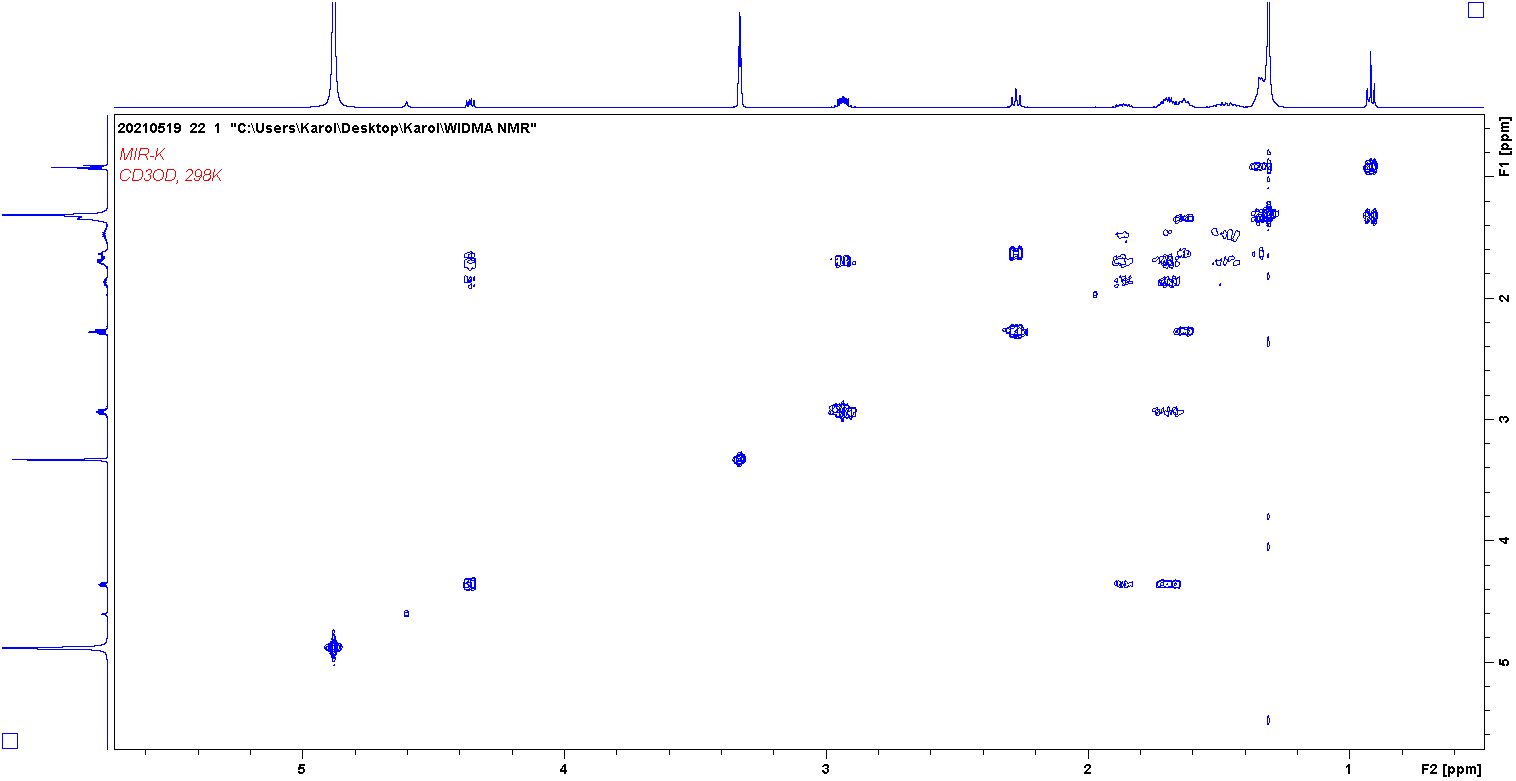
*

**Figure S3.** COSY spectrum of **1Mq**.

*
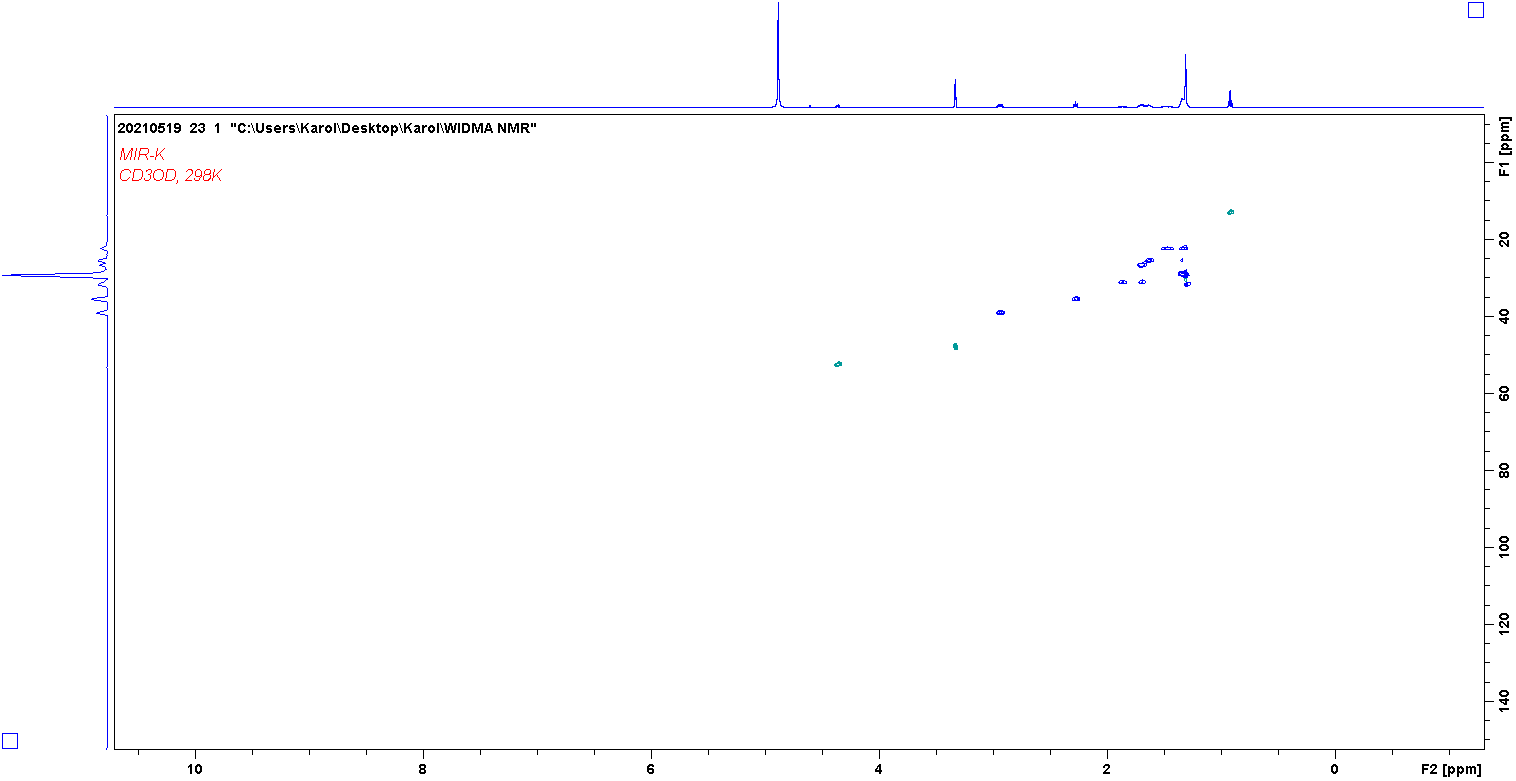
*

**Figure S4.** HSQC spectrum of **1Mq**.

*
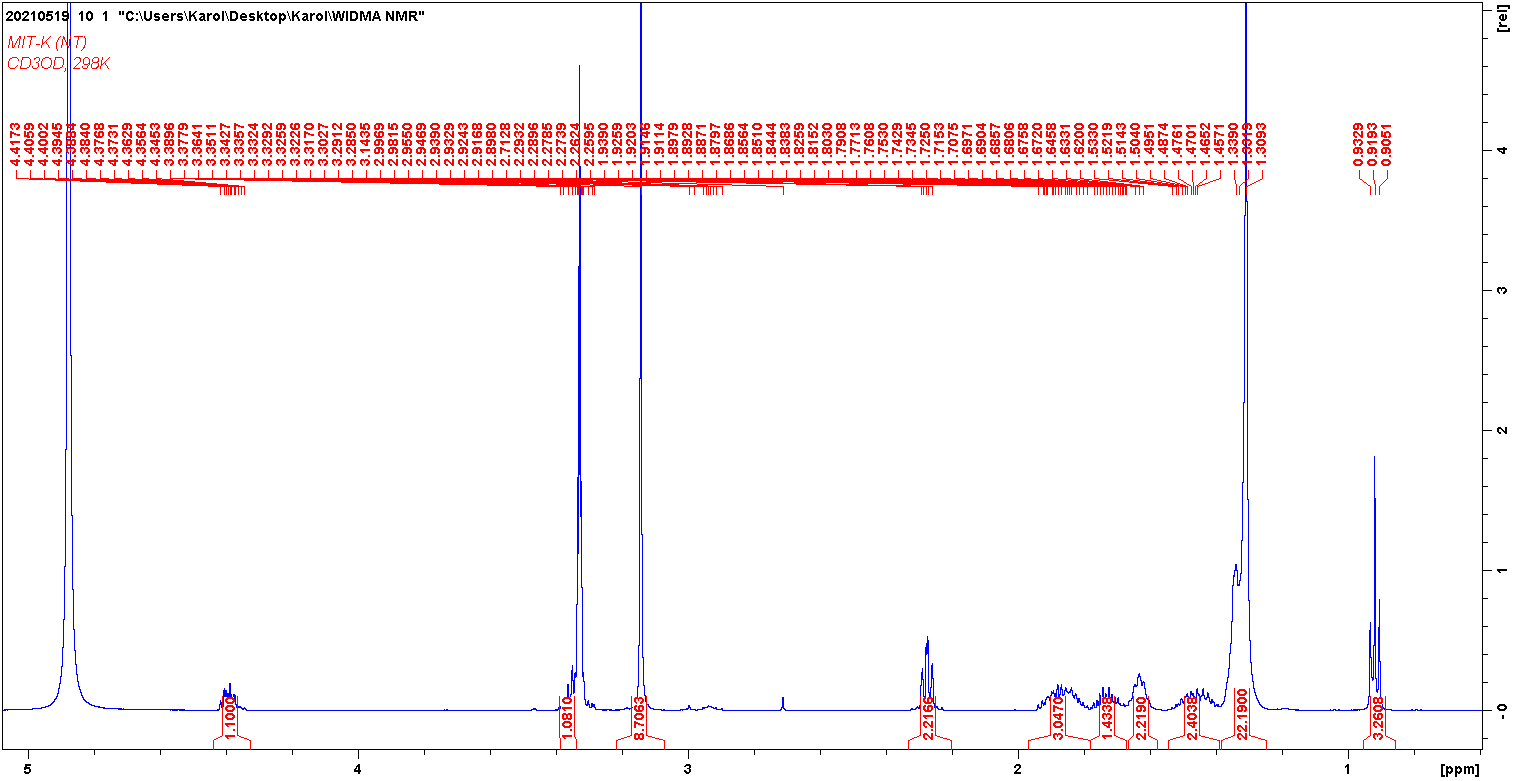
*

**Figure S5.** ^1^H NMR (CD_3_OD, 500 MHz) spectrum of **1Mq**.

*
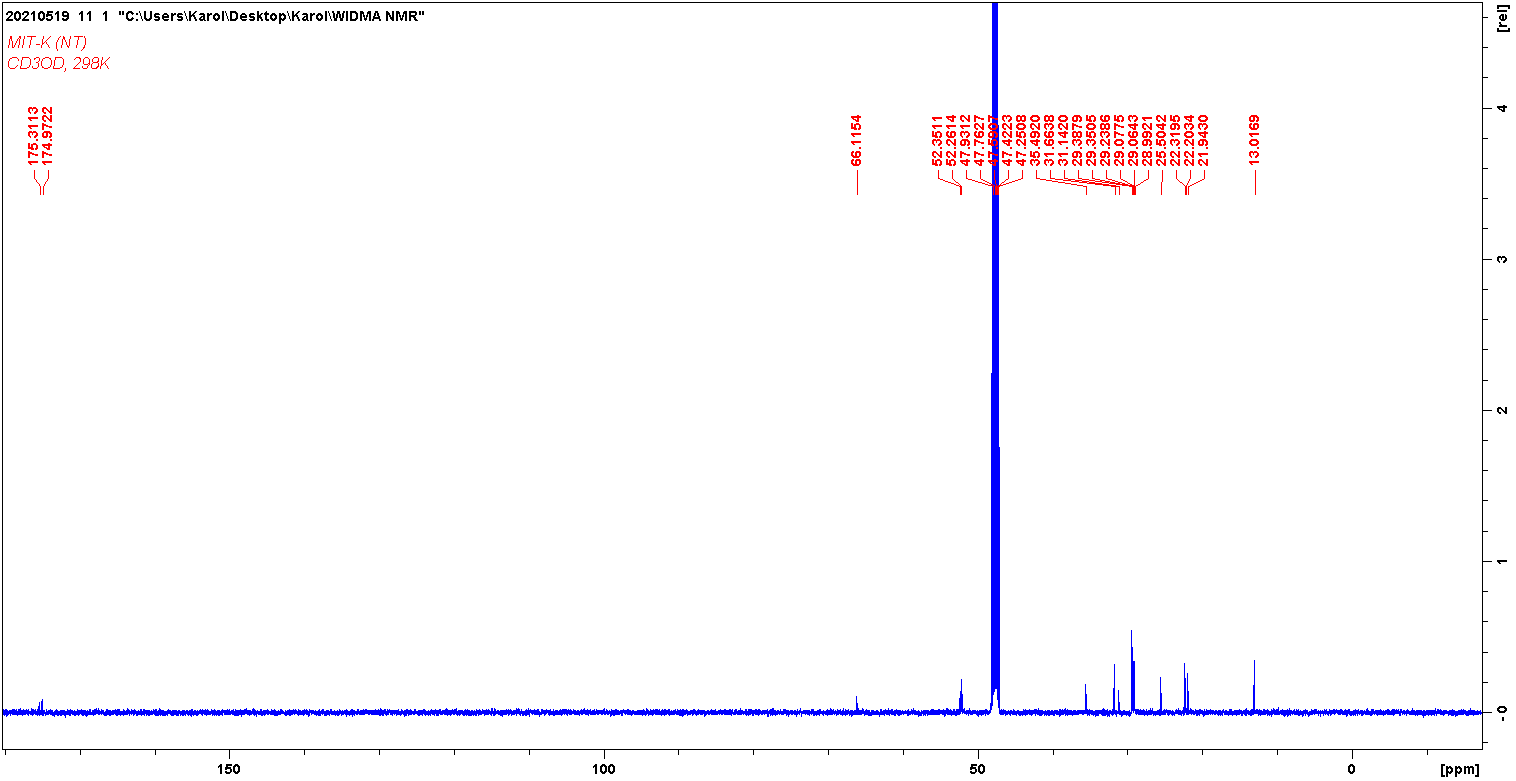
*

**Figure S6.** ^13^C NMR (CD_3_OD, 125 MHz) spectrum of **1Mq**.

*
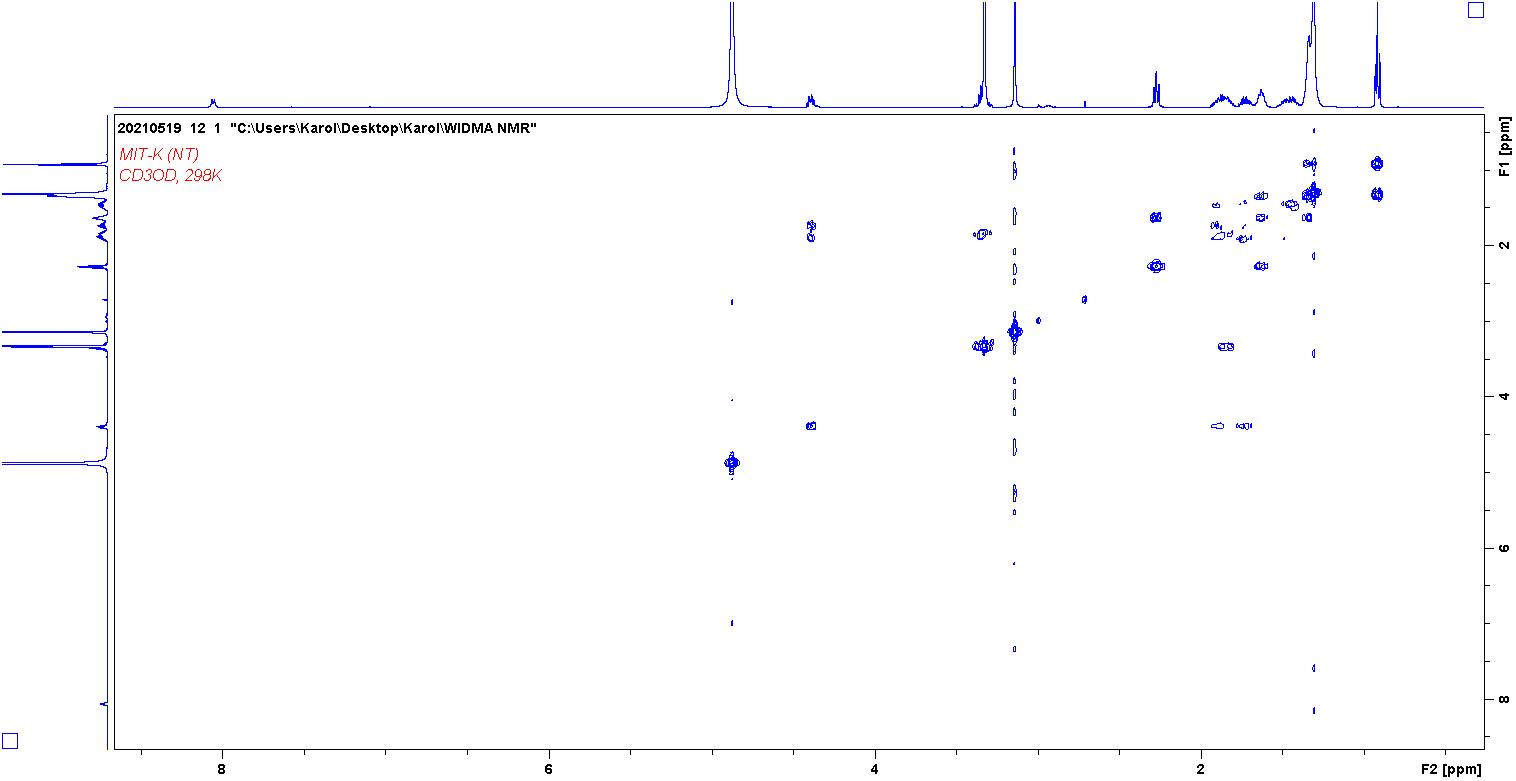
*

**Figure S7.** COSY spectrum of **1Mq**.

*
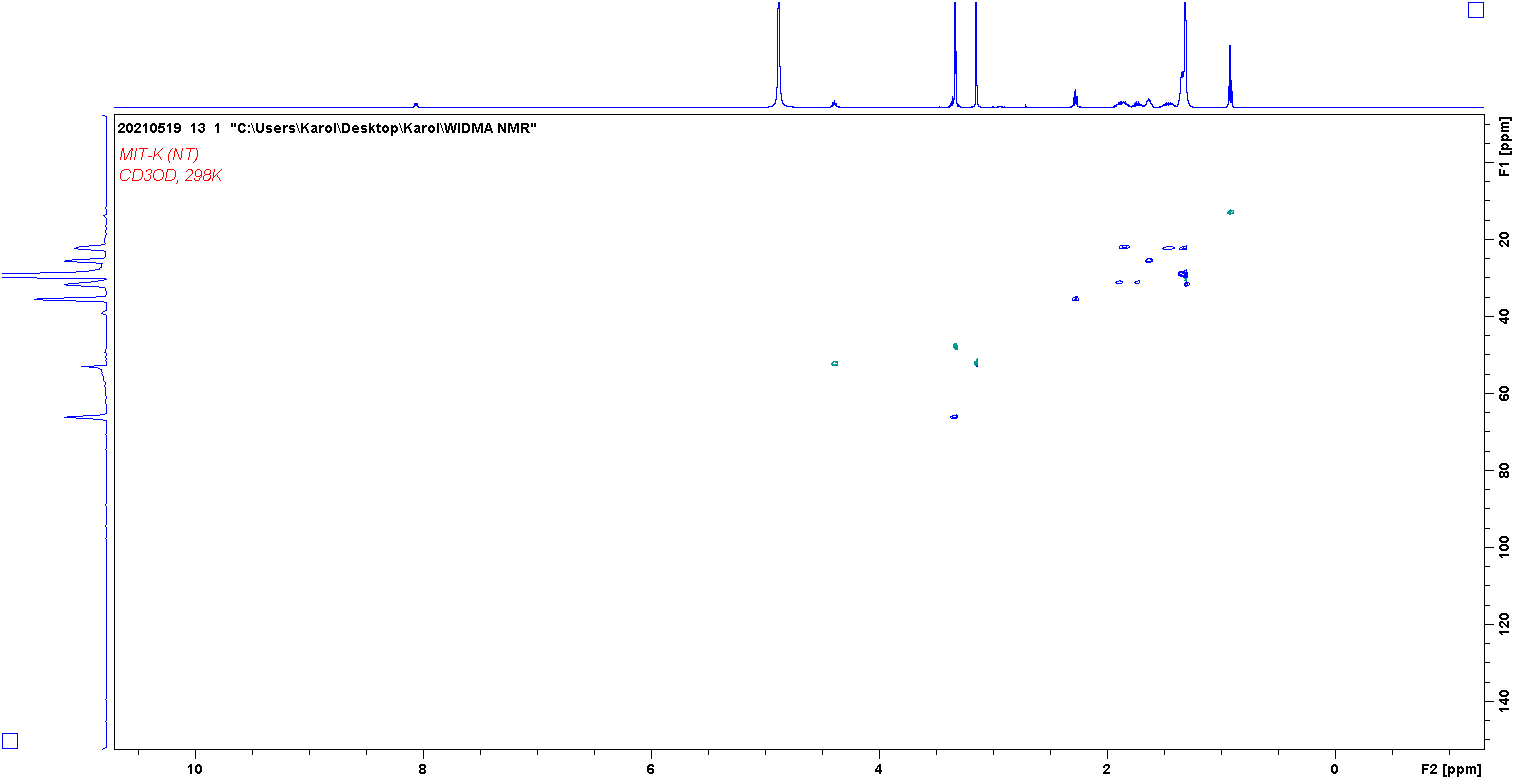
*

**Figure S8.** HSQC spectrum of **1Mq**.

# Retention time

**Figure S9**. Adjusted retention time (t’R) *vs.* number of amino acid residues.

Adjusted retention time of USCLs and qUSCLs with different fatty acid residues (M, Mq – C_14_, P, Pq – C_16_, S, Sq – C_18_) are correlated with number of amino acid residues – exponential function.

t’R = a(nAA)^b^

| Group | Function | R^2^ |
| --- | --- | --- |
| M | y = 19.478x^-0.305^ | 0.9983 |
| Mq | y = 20.166x^-0.311^ | 0.9997 |
| P | y = 24.103x^-0.319^ | 0.99996 |
| Pq | y = 24.53x^-0.329^ | 0.9982 |
| S | y = 28.472x^-0.34^ | 0.9992 |
| Sq | y = 29.598x^-0.342^ | 0.9997 |

Difference between adjusted retention times of qUSCLs and corresponding USCLs (Δt'R) are correlated with number of amino acid residues – quadratic function.

Δt'R = a(nAA)^2^ + b(nAA) + c

**Figure S10**. Difference in adjusted retention time (t’R) *vs.* number of amino acid residues.

| Group | Function | R^2^ |
| --- | --- | --- |
| qM-M | y = -0.0485x^2^ + 0.1239x + 0.5335 | 0.9962 |
| qP-P | y = -0.0582x^2^ + 0.1621x + 0.2278 | 0.6718 |
| qS-S | y = -0.0288x^2^ - 0.0095x + 1.0902 | 0.9133 |

# HC_50_ *vs.* hydrophobicity

HC_50_ is correlated with tR – exponential function.

HC_50_ = a(t’R)^b^

**Figure S11.** HC_50_ *vs.* adjusted retention time (t’R).

| Group | Function | R^2^ |
| --- | --- | --- |
| USCLs | y = 3×10^11^×(tR)^-7.612^ | 0.8549 |
| qUSCLs | y = 3×10^10^×(tR)^-6.451^ | 0.6993 |

Peptide **1M** was intentionally excluded from USCLs series (green triangle). Including **1M** into USCLs series effect in diminished correlation – R^2^ of 0.354.

# Antimicrobial activity – MIC values

**Table S1.** Antimicrobial activity (MIC [µg/mL]) of the lipopeptides against planktonic cells

| **Peptide** | **Fatty acid** | ***E.***  ***faecium***  ***ATCC***  ***700221*** | ***S.***  ***aureus***  **ATCC**  **33591** | ***K.***  ***pneumoniae***  **ATCC**  **700603** | ***A.***  ***baumannii***  **ATCC**  **BAA 1605** | ***P.***  ***aeruginosa***  **ATCC**  **9027** | ***K.***  ***aerogenes***  **ATCC**  **13048** | ***C.***  ***glabrata***  **ATCC**  **15126** |
| --- | --- | --- | --- | --- | --- | --- | --- | --- |
| **1M** | **C_14_** | 16 | 16 | >512 | >512 | >512 | >512 | >512 |
| **2M** |  | 32 | 16 | >512 | 512 | 128 | 512 | 256 |
| **3M** |  | 32 | 64 | >512 | 512 | 128 | 256 | 512 |
| **4M** |  | 16 | 32 | >512 | 512 | 256 | 256 | 512 |
| **1P** | **C_16_** | 4 | >512 | >512 | >512 | >512 | >512 | >512 |
| **2P** |  | 8 | 4 | 128 | 64 | 16 | 32 | 16 |
| **3P** |  | 8 | 8 | 32 | 64 | 32 | 8 | 64 |
| **4P** |  | 4 | 8 | 32 | 64 | 32 | 8 | 64 |
| **1S** | **C_18_** | >512 | >512 | >512 | >512 | >512 | >512 | >512 |
| **2S** |  | 4 | 2 | 64 | 32 | 128 | 32 | 8 |
| **3S** |  | 4 | 4 | 8 | 32 | 16 | 4 | 8 |
| **4S** |  | 4 | 8 | 8 | 16 | 8 | 4 | 16 |
| **Inactive compounds**  **(MIC >512 µg/mL)** | | 1 | 2 | 6 | 3 | 3 | 3 | 3 |
| **Mean MIC of USCLs** | | 12.0 | 16.2 | 45.3 | 200.9 | 82.7 | 123.6 | 161.8 |
| **1Mq** | **C_14_** | 32 | 32 | >512 | 128 | 128 | 256 | 32 |
| **2Mq** |  | 128 | 128 | >512 | >512 | >512 | >512 | 512 |
| **3Mq** |  | 256 | >512 | >512 | >512 | >512 | >512 | >512 |
| **4Mq** |  | 128 | >512 | >512 | >512 | >512 | >512 | >512 |
| **1Pq** | **C_16_** | 16 | 16 | 512 | 64 | >512 | 256 | 8 |
| **2Pq** |  | 16 | 16 | 512 | 128 | 512 | 512 | 64 |
| **3Pq** |  | 32 | 256 | >512 | >512 | >512 | 512 | >512 |
| **4Pq** |  | 32 | 512 | >512 | >512 | >512 | 512 | >512 |
| **1Sq** | **C_18_** | 4 | 8 | >512 | >512 | >512 | >512 | 4 |
| **2Sq** |  | 16 | 8 | 128 | 64 | >512 | 64 | 16 |
| **3Sq** |  | 16 | 64 | 256 | 128 | 512 | 64 | 128 |
| **4Sq** |  | 16 | 64 | 256 | 256 | 512 | 64 | 512 |
| **Inactive compounds**  **(MIC >512 µg/mL)** | | 0 | 2 | 7 | 6 | 8 | 4 | 4 |
| **Mean MIC of qUSCLs** | | 57.7 | 110.4 | 332.8 | 128.0 | 416.0 | 280.0 | 159.5 |
| **BAC** | | 4 | 4 | 16 | 16 | 32 | 16 | 4 |

BAC – benzalkonium chloride

# Antimicrobial activity vs. hydrophobicity

## 4.1. E. faecium ATCC 700221, USCLs

**Figure S12.** Log_2_MIC of USCLs against *E. faecium* ATCC 700221 *vs.* t’R.

## 4.2. E. faecium ATCC 700221, qUSCLs

**Figure S13.** Log_2_MIC of qUSCLs against *E. faecium* ATCC 700221 *vs.* t’R.

## 4.3. S. aureus ATCC 33591, USCLs

**Figure S14.** Log_2_MIC of USCLs against *S. aureus* ATCC 33591 *vs.* t’R.

## 4.4. S. aureus ATCC 33591, qUSCLs

**Figure S15.** Log_2_MIC of qUSCLs against *S. aureus* ATCC 33591 *vs.* t’R.

## 4.5. K. pneumoniae ATCC 700603, USCLs

**Figure S16.** Log_2_MIC of USCLs against *K. pneumoniae* ATCC 700603 *vs.* t’R.

## 4.6. K. pneumoniae ATCC 700603, qUSCLs

**Figure S17.** Log_2_MIC of qUSCLs against *K. pneumoniae* ATCC 700603 *vs.* t’R.

## 4.7. A. baumannii ATCC BAA 1605, USCLs

**Figure S18.** Log_2_MIC of USCLs against *A. baumannii* ATCC BAA 1605 *vs.* t’R.

## 4.8. A. baumannii ATCC BAA 1605, qUSCLs

**Figure S19.** Log_2_MIC of qUSCLs against *A. baumannii* ATCC BAA 1605 *vs.* t’R.

## 4.9. P. aeruginosa ATCC 9027, USCLs

**Figure S20.** Log_2_MIC of USCLs against *P. aeruginosa* ATCC 9027 *vs.* t’R.

## 4.10. P. aeruginosa ATCC 9027, qUSCLs

**Figure S21.** Log_2_MIC of qUSCLs against *P. aeruginosa* ATCC 9027 *vs.* t’R.

## 4.11. K. aerogenes ATCC 13048, USCLs

**Figure S22.** Log_2_MIC of USCLs against *K. aerogenes* ATCC 13048 *vs.* t’R.

## 4.12. K. aerogenes ATCC 13048, qUSCLs

**Figure S23.** Log_2_MIC of qUSCLs against *K. aerogenes* ATCC 13048 *vs.* t’R.

## 4.13. C. glabrata ATCC 15126, USCLs

**Figure S24.** Log_2_MIC of USCLs against *C. glabrata* ATCC 15126 *vs.* t’R.

## 4.14. C. glabrata ATCC 15126, qUSCLs

**Figure S25.** Log_2_MIC of qUSCLs against *C. glabrata* ATCC 15126 *vs.* t’R.

# IC_50_ *vs.* hydrophobicity

**Figure S26.** IC_50_ *vs.* adjusted retention time (t’R).

IC_50_ is correlated with t’R – expotential function.

# Selectivity indexes (SIs) – MIC and HC_50_ *vs.* t’R

## 6.1. MICs of USCLs against E. faecium ATCC 700221 and HC_50_ vs. hydrophobicity

**Figure S27.** SIs of USCLs (MICs – *E. faecium* and HC_50_) *vs.* adjusted retention time (t’R).

Peptide **1M** was intentionally excluded from USCLs series (green triangle). Including **1M** into USCLs series effect in diminished correlation – R^2^ of 0.0352.

## 6.2. MIC of qUSCLs against E. faecium ATCC 700221 and HC_50_ vs. hydrophobicity

**Figure S28.** SIs of qUSCLs (MICs – *E. faecium* and HC_50_) *vs.* adjusted retention time (t’R).

## 6.3. MIC of USCLs against S. aureus ATCC 33591 and HC_50_ vs. hydrophobicity

**Figure S29.** SIs of USCLs (MICs – *S. aureus* and HC_50_) *vs.* adjusted retention time (t’R).

Peptide **1M** was intentionally excluded from USCLs series (green triangle). Including **1M** into USCLs series effect in diminished correlation – R^2^ of 0.0516.

## 6.4. MIC of qUSCLs against S. aureus ATCC 33591 and HC_50_ vs. hydrophobicity

**Figure S30.** SIs of qUSCLs (MICs – *S. aureus* and HC_50_) *vs.* adjusted retention time (t’R).

## 6.5. MIC of USCLs against K. pneumoniae ATCC 700603 and HC_50_ vs. hydrophobicity

**Figure S31.** SIs of USCLs (MICs – *K. pneumoniae* and HC_50_) *vs.* adjusted retention time (t’R).

Red triangle – **4P** with SI > 16.

## 6.6. MIC of qUSCLs against K. pneumoniae ATCC 700603 and HC_50_ vs. hydrophobicity

**Figure S32.** SIs of qUSCLs (MICs – *K. pneumoniae* and HC_50_) *vs.* adjusted retention time (t’R).

## 6.7. MIC of USCLs against A. baumannii ATCC BAA 1605 and HC_50_ vs. hydrophobicity

**Figure S33.** SIs of USCLs (MICs – *A. baumannii* and HC_50_) *vs.* adjusted retention time (t’R).

Red triangles – **3M**, **4M**, and **4P** with SIs >1, >1, and >8, respectively.

## 6.8. MIC of qUSCLs against A. baumannii ATCC BAA 1605 and HC_50_ vs. hydrophobicity

**Figure S34.** SIs of qUSCLs (MICs – *A. baumannii* and HC_50_) *vs.* adjusted retention time (t’R).

Red triangle – **4Sq** with SI >2.

## 6.9. MIC of USCLs against P. aeruginosa ATCC 9027 and HC_50_ vs. hydrophobicity

**Figure S35.** SIs of USCLs (MICs – *P. aeruginosa* and HC_50_) *vs.* adjusted retention time (t’R).

Red triangles – **3M**, **4M**, **4P** with SIs >4, >2, >16, respectively.

## 6.10. MIC of qUSCLs against P. aeruginosa ATCC 9027 and HC_50_ vs. hydrophobicity

**Figure S36.** SIs of qUSCLs (MICs – *P. aeruginosa* and HC_50_) *vs.* adjusted retention time (t’R).

Red triangle – **4Sq** with SI >1.

## 6.11. MIC of USCLs against K. aerogenes ATCC 13048 and HC_50_ vs. hydrophobicity

**Figure S37.** SIs of USCLs (MICs – *K. aerogenes* and HC_50_) *vs.* adjusted retention time (t’R).

Red triangles – **3M, 4M, 4P** with SIs >2, >2, and >64, respectively.

## 6.12. MIC of qUSCLs against K. aerogenes ATCC 13048 and HC_50_ vs. hydrophobicity

**Figure S38.** SIs of qUSCLs (MICs – *K. aerogenes* and HC_50_) *vs.* adjusted retention time (t’R).

Red triangle – **4Sq** with SI >64.

## 6.13. MIC of USCLs against C. glabrata ATCC 15126 and HC_50_ vs. hydrophobicity

**Figure S39.** SIs of USCLs (MICs – *C. glabrata* and HC_50_) *vs.* adjusted retention time (t’R).

Red triangles – **3M**, **4M**, and **4P** with SIs >1, >1 and >8, respectively.

## 6.14. MIC of qUSCLs against C. glabrata ATCC 15126 and HC_50_ vs. hydrophobicity

**Figure S40.** SIs of qUSCLs (MICs – *C. glabrata* and HC_50_) *vs.* adjusted retention time (t’R).

Red triangles – **2Mq** and **4Sq** with SIs >1.

# Selectivity indexes (SIs) – MIC *vs.* IC_50_

## 7.1. MICs of USCLs against E. faecium ATCC 700221 and IC_50_ vs. Hydrophobicity

**A)**

B)

**Figure S41.** SIs of USCLs (MICs – *E. faecium* and IC_50_) *vs.* adjusted retention time (t’R). **A)** All data as one series; **B)** three series – M (C_14_), P (C_16_), S (C_18_).

## 7.2. MIC of qUSCLs against E. faecium ATCC 700221 and IC_50_ vs. hydrophobicity

**A)**

B)

**Figure S42.** SIs of qUSCLs (MICs – *E. faecium* and IC_50_) *vs.* adjusted retention time (t’R). **A)** All data as one series, Red triangles – **4Mq, 3Pq, 4Pq and 4Sq** with SIs >4, >16, >16, >32, respectively. **B)** six series – Mq, >Mq (C_14_), Pq, >Pq (C_16_), Sq, >Sq (C_18_). The “>” symbol indicate that real SI is higher than that calculated.

## 7.3. MIC of USCLs against S. aureus ATCC 33591 and IC_50_ vs. hydrophobicity

**A)**

**B)**

**Figure S43.** SIs of USCLs (MICs – *S. aureus* and IC_50_) *vs.* adjusted retention time (t’R). **A)** All data as one serie. **B)** three series – M (C_14_), P (C_16_), S (C_18_).

## 7.4. MIC of qUSCLs against S. aureus ATCC 33591 and IC_50_ vs. hydrophobicity

**A)**

**B)**

**Figure S44.** SIs of qUSCLs (MICs – *S. aureus* and IC_50_) *vs.* adjusted retention time (t’R). **A)** All data as one series. Red tringles – **3Pq**, **4Pq**, **4Sq**, with SIs >2, >1, and >8, respectively. **B)** six series – Mq, >Mq (C_14_), Pq, >Pq (C_16_), Sq, >Sq (C_18_). The “>” symbol indicate that real SI is higher than that calculated.

## 7.5. MIC of USCLs against K. pneumoniae ATCC 700603 and IC_50_ vs. hydrophobicity

**Figure S45.** SIs of USCLs (MICs – *K. pneumoniae* and IC_50_) *vs.* adjusted retention time (t’R).

## 7.6. MIC of qUSCLs against K. pneumoniae ATCC 700603 and IC_50_ vs. hydrophobicity

**Figure S46.** SIs of qUSCLs (MICs – *K. pneumoniae* and IC_50_) *vs.* adjusted retention time (t’R).

**4Sq** has SI >2.

## 7.7. MIC of USCLs against A. baumannii ATCC BAA 1605 and IC_50_ vs. hydrophobicity

**Figure S47.** SIs of USCLs (MICs – *A. baumannii* and IC_50_) *vs.* adjusted retention time (t’R).

## 7.8. MIC of qUSCLs against A. baumannii ATCC BAA 1605 and IC_50_ vs. hydrophobicity

**Figure S48.** SIs of qUSCLs (MICs – *A. baumannii* and IC_50_) *vs.* adjusted retention time (t’R).

## 7.9. MIC of USCLs against P. aeruginosa ATCC 9027 and IC_50_ vs. hydrophobicity

**Figure S49.** SIs of USCLs (MICs – *P. aeruginosa* and IC_50_) *vs.* adjusted retention time (t’R).

## 7.10. MIC of qUSCLs against P. aeruginosa ATCC 9027 and IC_50_ vs. hydrophobicity

**Figure S50.** SIs of qUSCLs (MICs – *P. aeruginosa* and IC_50_) *vs.* adjusted retention time (t’R).

**4Sq** has SI > 1.

## 7.11. MIC of USCLs against K. aerogenes ATCC 13048 and IC_50_ vs. hydrophobicity

**Figure S51.** SIs of USCLs (MICs – *K. aerogenes* and IC_50_) *vs.* adjusted retention time (t’R).

## 7.12. MIC of qUSCLs against K. aerogenes ATCC 13048 and IC_50_ vs. hydrophobicity

**Figure S52.** SIs of qUSCLs (MICs – *K. aerogenes* and IC_50_) *vs.* adjusted retention time (t’R).

**4Sq** has SI > 8.

## 7.13. MIC of USCLs against C. glabrata ATCC 15126 and IC_50_ vs. hydrophobicity

**Figure S53.** SIs of USCLs (MICs – *C. glabrata* and IC_50_) *vs.* adjusted retention time (t’R).

## 7.14. MIC of qUSCLs against C. glabrata ATCC 15126 and IC_50_ vs. hydrophobicity

**Figure S54.** SIs of qUSCLs (MICs – *C. glabrata* and IC_50_) *vs.* adjusted retention time (t’R).

**4Sq** has SI > 1.

# Comparison SIs between analogs

To evaluate effect of quaternization on peptides selectivity particular SIs of qUSCLs were divided by SIs of parent USCLs.

**Table S2.** The quotients of SI_qUSCLs_/SI_USCLs_ in terms of HC_50_

| ***Equation*** | ***E. faecium*** | ***S. aureus*** | ***K. pneumoniae*** | ***A. baumannii*** | ***P. aeruginosa*** | ***K. aerogenes*** | ***C. glabrata*** |
| --- | --- | --- | --- | --- | --- | --- | --- |
| SI_1Mq_/SI_1M_ | 0.1 | 0.1 | - | - | - | - | - |
| SI_2Mq_/SI_2M_ | - | - | - | - | - | - | 0.7 |
| SI_3Mq_/SI_3M_ | - | - | - | - | - | - | - |
| SI_4Mq_/SI_4M_ | - | - | - | - | - | - | - |
| SI_1Pq_/SI_1P_ | - | - | - | - | - | - | - |
| SI_2Pq_/SI_2P_ | 1.2 | 0.6 | 0.6 | 1.2 | 0.1 | 0.2 | 0.6 |
| SI_3Pq_/SI_3P_ | - | - | - | - | - | - | - |
| SIP_4Pq_/SI_4P_ | - | - | - | - | - | - | - |
| SI_1Sq_/SI_1S_ | - | - | - | - | - | - | - |
| SI_2Sq_/SI_2S_ | 0.3 | 0.3 | 0.5 | 0.5 |  | 0.5 | 0.5 |
| SI_3Sq_/SI_3S_ | 3.0 | 0.7 | 0.4 | 3.0 | 0.4 | 0.7 | 0.8 |
| SI_4Sq_/SI_4S_ | - | - | 0.3 | 0.7 | 0.2 | 0.7 | 0.3 |

* green shading indicate on more selective qUSCLs

**Table S3.** The quotients of SI_qUSCLs_/SI_USCLs_ in terms of IC_50_

| ***Equation*** | ***E. faecium*** | ***S. aureus*** | ***K. pneumoniae*** | ***A. baumannii*** | ***P. aeruginosa*** | ***K. aerogenes*** | ***C. glabrata*** |
| --- | --- | --- | --- | --- | --- | --- | --- |
| SI_1Mq_/SI_1M_ | 2.9 | 2.9 | - | - | - | - | - |
| SI_2Mq_/SI_2M_ | 2.0 | 1.0 | - | - | - | - | 3.9 |
| SI_3Mq_/SI_3M_ | 0.3 | - | - | - | - | - | - |
| SI_4Mq_/SI_4M_ | >0.3 | - | - | - | - | - | - |
| SI_1Pq_/SI_1P_ | 2.7 | - | - | - | - | - | - |
| SI_2Pq_/SI_2P_ | 4.9 | 2.4 | 2.4 | 4.9 | 0.3 | 0.6 | 2.4 |
| SI_3Pq_/SI_3P_ | >2.8 | >0.3 | - | - | - | - | - |
| SIP_4Pq_/SI_4P_ | >0.5 | >0.1 | - | - | - | - | - |
| SI_1Sq_/SI_1S_ | - | - | - | - | - | - | - |
| SI_2Sq_/SI_2S_ | 4.5 | 4.5 | 8.7 | 8.8 | - | 8.8 | 9.1 |
| SI_3Sq_/SI_3S_ | 1.3 | 0.3 | 0.2 | 1.3 | 0.2 | 0.3 | 0.3 |
| SI_4Sq_/SI_4S_ | >0.8 | >0.4 | >0.1 | >0.2 | >0.1 | >0.2 | >0.1 |

* green shading indicate on more selective qUSCLs
